# Space charge regulation for ultra-stable all-solid-state lithium batteries by engineering of argyrodite electrolyte

**DOI:** 10.1093/nsr/nwag015

**Published:** 2026-01-10

**Authors:** Jingjing Wang, Linan Jia, Yibo Du, Bangjun Guo, Haozhe Geng, Qianjin Huang, Junbo Hou, Jinhui Zhu, Xiaodong Zhuang

**Affiliations:** The Soft2D Lab, State Key Laboratory of Synergistic Chem-Bio Synthesis, State Key Laboratory of Metal Matrix Composites, Shanghai Key Laboratory of Electrical Insulation and Thermal Ageing, Department of Polymer Science and Engineering, School of Chemistry and Chemical Engineering, Shanghai Jiao Tong University, Shanghai 200240, China; Department of Intelligent Vehicle Research, School of Mechanical Engineering, Shanghai Jiao Tong University, Shanghai 200240, China; Department of Intelligent Vehicle Research, School of Mechanical Engineering, Shanghai Jiao Tong University, Shanghai 200240, China; Department of Intelligent Vehicle Research, School of Mechanical Engineering, Shanghai Jiao Tong University, Shanghai 200240, China; The Soft2D Lab, State Key Laboratory of Synergistic Chem-Bio Synthesis, State Key Laboratory of Metal Matrix Composites, Shanghai Key Laboratory of Electrical Insulation and Thermal Ageing, Department of Polymer Science and Engineering, School of Chemistry and Chemical Engineering, Shanghai Jiao Tong University, Shanghai 200240, China; The Soft2D Lab, State Key Laboratory of Synergistic Chem-Bio Synthesis, State Key Laboratory of Metal Matrix Composites, Shanghai Key Laboratory of Electrical Insulation and Thermal Ageing, Department of Polymer Science and Engineering, School of Chemistry and Chemical Engineering, Shanghai Jiao Tong University, Shanghai 200240, China; Power System Resources Environmental Technology Co., Ltd., Jiaxing 314399, China; The Soft2D Lab, State Key Laboratory of Synergistic Chem-Bio Synthesis, State Key Laboratory of Metal Matrix Composites, Shanghai Key Laboratory of Electrical Insulation and Thermal Ageing, Department of Polymer Science and Engineering, School of Chemistry and Chemical Engineering, Shanghai Jiao Tong University, Shanghai 200240, China; The Soft2D Lab, State Key Laboratory of Synergistic Chem-Bio Synthesis, State Key Laboratory of Metal Matrix Composites, Shanghai Key Laboratory of Electrical Insulation and Thermal Ageing, Department of Polymer Science and Engineering, School of Chemistry and Chemical Engineering, Shanghai Jiao Tong University, Shanghai 200240, China; Frontiers Science Center for Transformative Molecules, Zhanjiang Institute for Advanced Study, Shanghai Jiao Tong University, Shanghai 201203, China

**Keywords:** WO_3_ substitution, argyrodite electrolyte, all-solid-state Li batteries, space charge layer

## Abstract

Space charge layer (SCL) formation at cathode–electrolyte interfaces severely limits the performance of sulfide-based all-solid-state Li batteries (ASSLBs). While conventional strategies focus on cathode modifications, we propose a novel electrolyte-centric approach by incorporating WO_3_ into argyrodite electrolyte (Li_5.49_P_0.99_W_0.01_S_4.47_O_0.03_Cl_1.5_). This design achieves a record-high ionic conductivity of 13.5 mS cm^−1^ (at 25°C) among O-substituted argyrodites. When paired with a LiNi_0.92_Co_0.05_Mn_0.03_O_2_ cathode, the full cell delivers 217 mAh g^−1^ at 0.1C, and retains 92% capacity after 1000 cycles (1C) and 80% capacity after 5000 cycles (5C), far outperforming the cells with frequently-used Li_5.5_PS_4.5_Cl_1.5_ argyrodite electrolytes (200 mAh g^−1^ at 0.1C; failure at 408 cycles at 1C). Mechanistic studies reveal that WO_3_ substitution modulates the electrolyte’s chemical potential to align with the cathode, reducing interfacial energy barriers and inhibiting Li^+^ depletion, and then significantly suppresses SCL effects. This work pioneers an electrolyte engineering strategy to mitigate SCL issues, enabling high-energy-density, ultra-stable ASSLBs.

## Teaser

Introducing WO3 into argyrodite electrolyte aligns its chemical potential with the cathode and suppresses space charge effects, enabling highly stable all-solid-state lithium batteries.

## INTRODUCTION

All-solid-state Li batteries (ASSLBs) represent a transformative advancement in energy storage technology, with potential applications ranging from portable electronics to electric vehicles and aerial mobility systems. By replacing flammable liquid electrolytes with inorganic solid electrolytes (SEs), ASSLBs address critical safety concerns while offering higher energy densities than conventional Li-ion batteries [1–3]. Among various SE materials, sulfide-based SEs (SSEs) have emerged as particularly promising candidates due to their exceptional room-temperature ionic conductivity (${{\mathrm{\sigma }}}_i$ >10 mS cm^−1^) and mechanical flexibility, outperforming oxide-, polymer-, and halide-based alternatives [4–6]. Despite these advantages, the development of sulfide-based ASSLBs faces significant challenges, particularly at the cathode–electrolyte interface [7–9]. Key issues include (i) chemical/electrochemical instability, (ii) poor solid–solid contact, and (iii) space charge layer (SCL) formation [10,11]. While extensive research has focused on mitigating interfacial reactions and improving physical contact [12], the SCL effect—a critical yet often overlooked phenomenon—remains insufficiently addressed.

The SCL arises from a Li^+^ chemical potential mismatch between cathode active materials (CAMs) and SSEs, creating localized charge imbalance at their interface [13]. This effect depletes Li^+^ concentration near the interface, increasing ion migration barriers and interfacial resistance. SSEs are especially susceptible due to their low oxidation potentials and poor compatibility with high-voltage oxide cathodes. Current SCL mitigation strategies predominantly employ CAM-side modifications, typically through ion-conductive coatings (e.g. LiNbO_3_ [14], Li_1.4_Al_0.4_Ti_1.6_(PO_4_)_3_ [15], Li_3_BO_3_ [16,17], and Li_4_Ti_5_O_12_ [18]) or dielectric coatings (e.g. BaTiO_3_) [19]. These nanoscale coatings (1–5 nm) physically isolate the CAM from SSE while providing Li^+^ transport pathways, thereby suppressing SCL formation [10]. For instance, LiNbO_3_ coatings effectively reduce interfacial energy barriers by modulating Li^+^ adsorption sites and chemical potential gradients [11]. Alternative approaches involving CAM stoichiometry adjustment have also demonstrated limited SCL mitigation [20]. However, existing strategies for SCL mitigation focus exclusively on modifying the CAM, overlooking the potential of SSE optimization. Current efforts in SSE development primarily center on atomic substitution to enhance ionic conductivity [21–25], air stability [26–28], and interfacial compatibility [29,30].

Herein, we synthesized a series of WO_3_-substituted, Cl-rich argyrodite SSEs using conventional high-energy ball milling followed by calcination. The optimal SSE (Li_5.49_P_0.99_W_0.01_S_4.47_O_0.03_Cl_1.5_) exhibits the highest ${{\mathrm{\sigma }}}_i$ of 13.5 mS cm^−1^ at 25°C among reported O-substituted argyrodite SSEs. When integrated with a Li–In anode and a LiNi_0.92_Co_0.05_Mn_0.03_O_2_ (NCM92) CAM, the resulting full cell delivers a high specific capacity of 217 mAh g^−1^ at 0.1C and maintains stable operation even at a high rate of 5C. Long-term cycling tests demonstrate excellent stability, with capacity retentions of 92% after 1000 cycles at 1C and 80% after 5000 cycles at 5C. By comparison, the full cell employing the basic SSE (i.e. Li_5.5_PS_4.5_Cl_1.5_) delivers a specific capacity of 200 mAh g^−1^ at 0.1C, but fails after only 408 cycles at 1C. Moreover, the Li/*μ*-Si|NCM92 practical pouch cell with optimal SSE achieves a high energy density of 363 Wh kg^−1^ and maintains stable performance over 100 cycles. A combination of *in situ* and *ex situ* characterizations reveals that, compared to the basic SSE/NCM92 system, the SCL effect is significantly suppressed at the interface between the optimal SSE and the NCM92. Furthermore, density functional theory (DFT) calculations show that the incorporation of WO_3_ modulates the chemical potential of the SSE, aligning its Fermi level more closely with that of the CAM. This alignment reduces the interfacial energy barrier and mitigates the SCL effect, thereby enhancing the overall electrochemical performance of the full cell.

## RESULTS AND DISCUSSION

### Synthesis of Li_5.5__−_*_x_*P_1__−_*_x_*W*_x_*S_4.5__−__3_*_x_*O_3_*_x_*Cl_1.5_ SSEs

**Figure 1.**
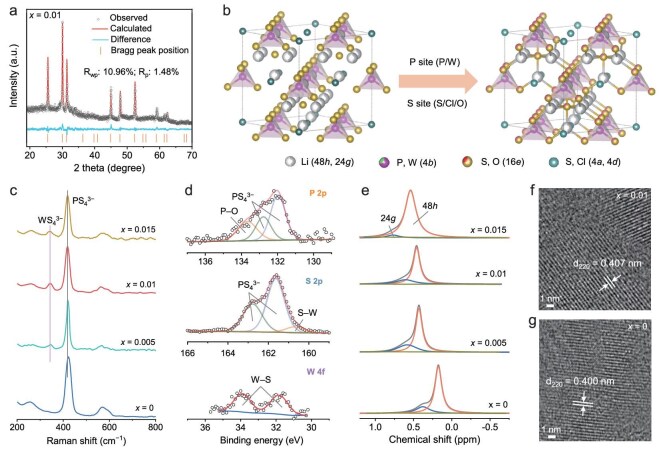
Design strategy and structural characterization of Li_5.5__−_*_x_*P_1__−_*_x_*W*_x_*S_4.5–3_*_x_*O_3_*_x_*Cl_1.5_ SSEs. (a) XRD patterns with Rietveld refinement for the sample with *x* = 0.01. (b) Schematic illustration of the WO_3_-substitution process. (c) Raman spectra of SSEs with *x* = 0, 0.005, 0.01, and 0.015. (d) P 2p, S 2p, and W 4f XPS spectra of sample with *x* = 0.01. (e) ^7^Li MAS ssNMR spectra for SSEs with varying W content (*x* = 0, 0.005, 0.01, 0.015). HRTEM images of the samples with (f) *x* = 0.01 and (g) *x* = 0.

The target WO_3_-substituted, Cl-rich argyrodite SSEs (Li_5.5−_*_x_*P_1−_*_x_*W*_x_*S_4.5−3_*_x_*O_3_*_x_*Cl_1.5_, *x* = 0, 0.005, 0.01, 0.015) were synthesized via conventional high-energy ball milling followed by thermal calcination, using stoichiometric mixtures of Li_2_S, P_2_S_5_, LiCl, and WO_3_ precursors (Fig. S1) [31]. X-ray diffraction (XRD) patterns confirm all synthesized SSEs adopt a cubic argyrodite structure (space group *F-*43*m*), as evidenced by their perfect match with Li_7_PS_6_ reference (PDF#34–0688, Fig. S2). Notably, systematic peak shifts toward lower angles (e.g. from 30.22° to 29.88°) with increasing WO_3_ content quantitatively demonstrate unit cell expansion—a direct consequence of substituting smaller P^5+^ (38 pm) with larger W^6+^ (62 pm) cations. Rietveld refinement of a selective composition (*x* = 0.01) reveals enlarged lattice parameters (*a* = *b* = *c* = 9.876 Å) compared to the basic SSE (*x* = 0, 9.789 Å, Fig. 1a and Fig. S3, Tables S1 and S2) [32]. This structural expansion is expected to enhance Li^+^ mobility through widened ionic transport pathways. The refined structural model (Fig. 1b) shows W atoms preferentially occupy 4*b* sites (P positions) forming WS_4_^3−^ tetrahedra, while O substitutes S at 16*e* sites.

Raman spectra (Fig. 1c) of synthesized SSEs exhibit characteristic PS_4_^3−^ vibrations (419–422 cm^−1^) confirming the argyrodite framework. The red-shift of this mode in spectra of WO_3_-substituted samples versus pristine reflects modified P–S bond dynamics due to lattice strain induced by W incorporation. The emergence of a new vibration at 343 cm^−1^ for spectra of WO_3_-containing SSEs, attributed to WS_4_^3−^ groups, provides direct evidence of successful W doping [32]. No residual WO_3_ can be observed in the WO_3_-substituted SSEs (Fig. S4). X-ray photoelectron spectroscopy (XPS) was employed to characterize the synthesized SSEs (Fig. 1d and Fig. S5). The P 2p, S 2p, and W 4f spectra of Li_5.49_P_0.99_W_0.01_S_4.47_O_0.03_Cl_1.5_ SSE exhibit distinct peaks corresponding to P–O (133.7 eV), S–W (160.7 eV), and W–S bonds (31.8 and 33.9 eV), respectively [33]. These P–O bonds originate from the partial substitution of S by O in the PS_4_^3−^ groups, or trace phosphates (decomposition products). In comparison, the P 2p and S 2p XPS spectra revealed that only PS_4_^3−^ can be observed in the basic SSE. These features confirm the successful incorporation of both W and O into the crystal lattice of the WO_3_-substituted SSEs.

The structural disorder and Li^+^ dynamics of the synthesized SSEs were investigated using ^7^Li magic-angle spinning solid-state nuclear magnetic resonance (MAS ssNMR) spectroscopy. As shown in Fig. 1e, two distinct resonant peaks corresponding to the 24*g* and 48*h* positions of Li are observed in the NMR spectra of all SSEs [34]. Notably, the ^7^Li NMR signals of WO_3_-substituted SSEs shift to low field compared to the WO_3_-free counterpart. This shift can be attributed to two main factors: (i) the incorporation of W atoms induces lattice expansion and facilitates Li^+^ migration. This may allow more Li^+^ to enter the lattice, thereby increasing the local Li^+^ concentration and resulting in a downfield shift of the resonance peak [6]. (ii) The partial substitution of S^2−^ by O^2−^ increases the local charge density in the Li^+^ environment, enhancing the electrostatic interactions between mobile Li^+^ and the anionic framework. This stronger interaction increases the shielding effect on Li^+^, contributing further to the observed chemical shift [35].

The morphology and elemental distribution of the synthesized SSEs were examined using scanning electron microscopy (SEM) and high-angle annular dark-field scanning transmission electron microscopy (HAADF-STEM). The SEM and HAADF-STEM images reveal a uniform distribution of P, S, Cl, W, and O elements across the samples (Figs S6–S8). Additionally, high-resolution TEM (HRTEM) was employed to evaluate the lattice parameters of the synthesized SSEs. For Li_5.49_P_0.99_W_0.01_S_4.47_O_0.03_Cl_1.5_ SSE, lattice fringes with spacings of 0.407 nm, corresponding to the (220) crystal plane, were observed (Fig. 1f). This value is larger than the 0.400 nm spacing (associated with the (220) plane) observed in the basic SSE (Fig. 1g), indicating unit cell expansion upon WO_3_ incorporation—consistent with the XRD results [36].

### Electrochemical properties of Li_5.5__−_*_x_*P_1__−_*_x_*W*_x_*S_4.5__−__3_*_x_*O_3_*_x_*Cl_1.5_ SSEs

**Figure 2.**
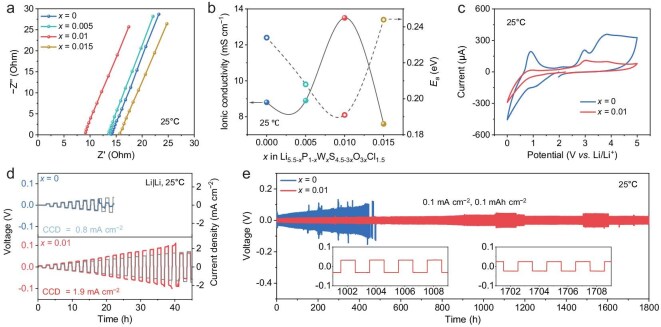
Electrochemical characterizations of Li_5.5−_*_x_*P_1−_*_x_*W*_x_*S_4.5−3_*_x_*O_3_*_x_*Cl_1.5_ SSEs. (a) Nyquist plots of SSEs (*x* = 0, 0.005, 0.01, and 0.015) obtained from EIS tests. (b) ${{\mathrm{\sigma }}}_i$ (at 25°C) and ${E}_a$ of SSEs (*x* = 0, 0.005, 0.01, and 0.015). (c) CV curves of SSEs (*x* = 0 and 0.01) measured over a voltage range of 0–5 V vs. Li/Li^+^. (d) CCDs of Li|Li symmetric cells with SSEs (*x* = 0 and 0.01). (e) Voltage profile of Li|Li symmetric cells using SSEs (*x* = 0 and 0.01), cycled at 0.1 mA cm^−2^ and 0.1 mAh cm^−2^ at 25°C. Insets: Enlarged voltage profiles showing stable Li plating/stripping behavior.

The ${{\mathrm{\sigma }}}_i$ of the synthesized Li_5.5−_*_x_*P_1−_*_x_*W*_x_*S_4.5−3_*_x_*O_3_*_x_*Cl_1.5_ SSEs was measured using electrochemical impedance spectroscopy (EIS). From the Nyquist plots (Fig. 2a), ${{\mathrm{\sigma }}}_i$ values (at 25°C) were calculated to be 8.8, 8.9, 13.5, and 7.6 mS cm^−1^ for SSEs with *x* = 0, 0.005, 0.01, and 0.015, respectively (Fig. 2b and Table S3). Notably, the optimal composition (*x* = 0.01) exhibits a ${{\mathrm{\sigma }}}_i$ as high as 13.5 mS cm^−1^, outperforming previously reported O-containing argyrodite electrolytes (Table S4), despite the general tendency of O substitution to reduce ${{\mathrm{\sigma }}}_i$ [37]. The activation energy (${E}_a$) of Li^+^ transport was also derived from the EIS data (Fig. S9) and corresponding Arrhenius plots (Fig. S10). An inverse relationship between ${E}_a$ and ${{\mathrm{\sigma }}}_i$ was observed as WO_3_ content increased (Table S5), with the optimal SSE exhibiting the lowest ${E}_a$ of 0.19 eV (Fig. 2b).

The substitution of P^5+^ (38 pm) by the larger W^6+^ (62 pm) induces lattice expansion, as confirmed by XRD and HRTEM results. This expansion widens the Li^+^ migration pathways and reduces the activation energy for Li^+^ transport. In addition, the introduction of W^6+^ introduces extra positive charge, which is compensated by the formation of Li^+^ vacancies, thereby further enhancing ionic mobility. In contrast, the substitution of S^2−^ by O^2−^ generally reduces ${{\mathrm{\sigma }}}_i$ due to the higher electronegativity and lower polarizability of O^2−^, stronger Li^+^–O binding, and associated lattice contraction. However, in our system, the presence of W^6+^ counteracts this negative effect. The formation of W–S bonds helps stabilize the structure and modulates the local electronic environment, reducing electron density on S and weakening Li^+^–S interactions, which in turn facilitates Li^+^ hopping. Thus, the cooperative interaction between W^6+^ and O^2−^ effectively mitigates the detrimental impact of O substitution and leads to an overall improvement in ${{\mathrm{\sigma }}}_i$.

Moreover, the incorporation of WO_3_ directly influences the electronic structure of the argyrodite SSE. Changes near the Fermi level can alter the material’s work function and its electron-donating/accepting tendency, which is intrinsically connected to its electrochemical stability and interfacial behavior.

In addition to ${{\mathrm{\sigma }}}_i$, electronic conductivity (${{\mathrm{\sigma }}}_e$) is a critical parameter for evaluating the electrochemical performance of SSEs, with low ${{\mathrm{\sigma }}}_e$ being desirable to suppress internal short circuits and enhance cell safety [35,38]. The ${{\mathrm{\sigma }}}_e$ of the optimal SSE was measured to be 4.58 × 10^−9^ S cm^−1^, which is significantly lower than that of the basic SSE (6.87 × 10^−8^ S cm^−1^) (Fig. S11 and Table S6), suggesting enhanced dendrite suppression capability for this WO_3_-substituted SSE. The electrochemical stability of the SSEs was assessed via cyclic voltammetry (CV) tests [39]. As shown in Fig. 2c, the optimal SSE exhibited weaker and broader redox peak intensities across a broad voltage range (0–5 V vs. Li/Li⁺) compared to the basic SSE. Given its higher ${{\mathrm{\sigma }}}_i$ and lower ${{\mathrm{\sigma }}}_e$, this indicates suppressed parasitic decomposition reactions, reflecting improved electrochemical stability of the optimal SSE.

To evaluate compatibility with Li metal anodes (LMAs), Li|Li symmetric cells with synthesized SSEs were assembled and tested at 25°C. Critical current density (CCD) measurements revealed that the optimal SSE-based cell achieved a highest CCD of 1.9 mA cm^−2^, more than double that of the WO_3_-free counterpart (0.8 mA cm^−2^) (Fig. 2d and Fig. S12). This indicates enhanced dendrite suppression and better interfacial compatibility with LMA for the optimal SSE. Furthermore, Li plating/stripping cycling performance was evaluated. At 0.1 mA cm^−2^ and 0.1 mAh cm^−2^, the Li|Li cell with optimal SSE maintained stable cycling for >1800 h with negligible polarization increase (Fig. 2e). Under harsher conditions (0.5 mA cm^−2^, 0.5 mAh cm^−2^), it remained stable for >700 h (Fig. S13). We noticed minor fluctuations in polarization voltage, which are attributed to dynamic interface evolution. In contrast, the cell with basic SSE showed a sudden increase in polarization voltage and short-circuited after only 445 h under the same low-current cycling conditions, indicating inferior dendrite suppression [39,40]. These results collectively demonstrate that the Li_5.49_P_0.99_W_0.01_S_4.47_O_0.03_Cl_1.5_ SSE offers superior compatibility with LMA, owing to its high ${{\mathrm{\sigma }}}_i$, low ${{\mathrm{\sigma }}}_e$, and excellent electrochemical stability.

### Cell performance of Li_5.5__−_*_x_*P_1__−_*_x_*W*_x_*S_4.5__−__3_*_x_*O_3_*_x_*Cl_1.5_ SSEs

**Figure 3.**
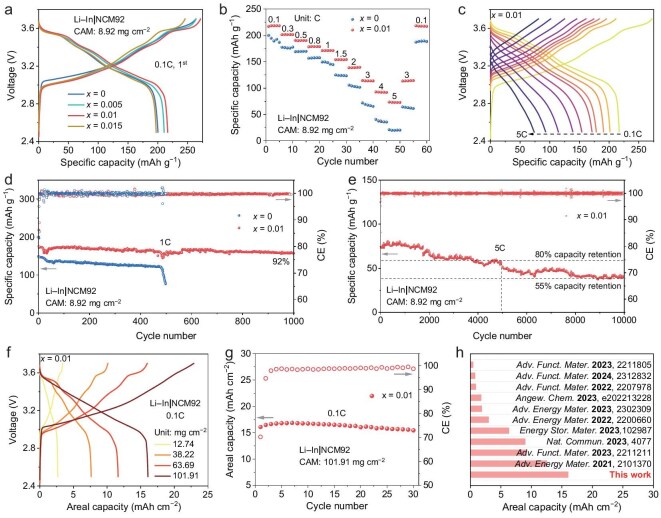
Electrochemical performance of Li_5.5−_*_x_*P_1−_*_x_*W*_x_*S_4.5−3_*_x_*O_3_*_x_*Cl_1.5_ SSE-based full cells. (a) Initial charge–discharge voltage profiles at 0.1C. (b) Rate capability across various current densities. (c) Voltage profiles under different C-rates. (d) Long-term cycling performance at 1C. (e) Extended cycling performance at 5C. (f) Voltage profiles at 0.1C with varying CAM mass loadings. (g) Cycling performance at 0.1C with a CAM mass loading of 101.91 mg cm^−2^. (h) Comparison of limited areal capacities with previously reported full cells.

To evaluate the electrochemical performance of the synthesized SSEs in ASSLBs, full cells comprising a Li–In alloy anode, Li_5.5−_*_x_*P_1−_*_x_*W*_x_*S_4.5−3_*_x_*O_3_*_x_*Cl_1.5_ SSE, and a NCM92 CAM (Fig. S14) were assembled (Fig. S15) and tested at 25°C under a pressure of 35 MPa. To enhance the uniformity and conductivity of the composite cathode, the SSEs used as catholytes were refined (Figs S16–S18). The NCM92 CAM had a mass loading of 8.92 mg cm^−2^. The initial charge–discharge profiles of full cells incorporating the four different SSE compositions are presented in Fig. 3a and Fig. S19. Notably, the cell utilizing the optimal SSE delivered the highest specific capacity of 217 mAh g^−1^ at 0.1C (1C = 200 mA g^−1^), with a Coulombic efficiency (CE) of 80%, surpassing the performance of cells with other SSE variants (e.g. 200 mAh g^−1^ at 0.1C for cell with basic SSE). We attribute the capacity difference between cells with optimal and basic SSEs primarily to the difference in ionic conductivity of the catholytes—higher ${{\mathrm{\sigma }}}_i$ helps reduce cell impedance and enhances charge-transfer kinetics (Fig. S20). Additionally, the superior chemical stability of the optimal SSE also contributes to improved cell performance. Subsequent rate capability assessments revealed that the full cell with the optimal SSE exhibited excellent performance across various current rates, delivering specific capacities of 217, 201, 190, 178, 171, 154, 139, 115, 93, and 74 mAh g^−1^ at rates of 0.1C, 0.3C, 0.5C, 0.8C, 1C, 1.5C, 2C, 3C, 4C, and 5C, respectively (Fig. 3b and c). In contrast, the cell employing the basic SSE demonstrated inferior rate capabilities, achieving specific capacities of 200, 176, 169, 157, 149, 125, 106, 71, 40, and 20 mAh g^−1^ under the same conditions (Fig. S21).

Long-term cycling stability tests further highlighted the superior performance of the optimal SSE-based full cell. At a rate of 1C, this cell maintained 92% of its initial capacity after 1000 cycles, whereas the basic SSE-based cell retained only 80% capacity after 408 cycles before rapid degradation ensued (Fig. 3d, Figs S22 and S23). The rapid capacity fade of this full cell is attributed to severe SCL formation, continuous interfacial decomposition, and mechanical cracking at the cathode–SSE interface. Even at elevated rates, the optimal SSE-based cell exhibited remarkable stability, retaining 88% capacity after 2000 cycles at 2C (Fig. S24) and sustaining 80% capacity over 5000 cycles at 5C (Fig. 3e). These findings highlight the superior electrochemical performance and cycling stability enabled by the optimally WO_3_-substituted SSE, surpassing both the WO_3_-free SSE and previously reported halogen-rich argyrodite SSEs (Table S7).

In addition, Li|NCM92 full cells employing the prepared SSEs were assembled and tested (Fig. S25). These cells exhibited significantly inferior performance compared to the Li–In|NCM92 full cells under identical conditions. This is attributed to the instability of the Li–SSE interface under harsh operating conditions (1.8 mA cm^−2^, 1.32 mAh cm^−2^), despite it demonstrated stability for >1800 h at 0.1 mA cm^−2^ and 0.1 mAh cm^−2^.

Recognizing the importance of CAM mass loading in determining the energy density of full cells, we investigated the performance of cells with varying CAM loadings using the optimal SSE. As depicted in Fig. 3f and Fig. S26, increasing the CAM mass loading from 12.74 to 101.91 mg cm^−2^ resulted in corresponding areal capacities of 2.7, 7.6, 11.7, and 16.1 mAh cm^−2^ at 0.1C. In comparison, the WO_3_-free SSE-based cell exhibited lower areal capacities under identical CAM loadings, with a limited areal capacity of only 11.6 mAh cm^−2^ (Fig. S27). Furthermore, the high-CAM-loading (101.91 mg cm^−2^) full cell with the optimal SSE demonstrated stable cycling over 30 cycles at 0.1C (Fig. 3g). A comparative analysis of limited areal capacities from recent studies (Fig. 3h) reveals that our work achieves a notable improvement, reaching 16.1 mAh cm^−2^, surpassing previously reported values for full cells utilizing similar anode and cathode configurations (Table S8) [32,41–48].

The observed enhancements in cell performance correlate positively with the ${{\mathrm{\sigma }}}_i$ of the employed SSEs. Higher ${{\mathrm{\sigma }}}_i$ facilitates more efficient Li^+^ transport, reducing cell resistance and polarization, thereby improving overall performance. This relationship is corroborated by differential capacity ($dQ/dV$) analyses, EIS, and galvanostatic intermittent titration technique (GITT) measurements. Across various cycling stages, cells with higher-conductivity SSEs exhibited smaller polarization between oxidation and reduction potentials (Fig. S28) and lower resistance values (Fig. S29 and Table S9) compared to those with lower-conductivity SSEs. GITT-derived Li^+^ diffusion coefficients (${D}_{{\mathrm{L}}{{\mathrm{i}}}^ + }$) further support these findings; the composite cathode with the optimal SSE exhibited the highest ${D}_{{\mathrm{L}}{{\mathrm{i}}}^ + }$ of 9.5 × 10^−8^ cm^2^ s^−1^, approximately two to four times greater than those of other synthesized SSE-based composite cathodes (Fig. S30).

Post-cycling analysis (100 cycles) confirmed that the optimal SSE maintained its structural integrity. The Raman spectrum of the optimal SSE showed no obvious impurity peaks (Fig. S31), and its S 2p and P 2p XPS spectra showed no signs of Li_2_S or Li_3_P. In contrast, the basic SSE exhibited clear signs of degradation, with Raman peaks indicating the presence of PO_4_^3−^ impurities (1050 cm^−1^) and cathode polarization (1341 cm^−1^). These findings were further corroborated by XPS analysis, which confirmed side products such as Li_2_S, PO_4_^3−^, and Li_3_P (Fig. S32) [49]. These results demonstrate that proper WO_3_ substitution enhances the SSE’s oxidation stability and its compatibility with the CAM.

### Analysis of the SCL effect

The SCL effect between the SSE and CAM was further explored to better understand the high performance of full cells using optimal WO_3_-substituted SSE. Initially, *in situ* EIS measurements and distribution of relaxation time (DRT) analysis were performed during the initial charging process of the full cells. As shown in Fig. S33, the Nyquist plots feature a high-frequency semicircle, a mid-frequency semicircle, and a low-frequency tail, which correspond to the anodic interfacial resistance (${R}_{a - i}$), cathodic interfacial resistance (${R}_{c - i}$), and Li^+^ diffusion resistance, respectively. For the full cell with the optimal SSE, the ${R}_{a - i}$ value increases gradually from 10.5 to 20.4 Ω during charging, while ${R}_{c - i}$ decreases from 64.5 to 48.4 Ω (Table S10). The increase in ${R}_{a - i}$ is attributed to undesirable side reactions at the anodic interface, while the decrease in ${R}_{c - i}$ is due to the formation of a gap-filling cathode electrolyte interphase (CEI), which enhances the contact between the SSE and CAM and facilitates Li^+^ transport. In contrast, the basic SSE-based full cell exhibits only slight variation in interfacial resistances at voltages below 3.4 V. However, these resistances sharply increase upon charging to 3.7 V (${R}_{a - i}$: 34.4 to 65.4 Ω; ${R}_{c - i}$: 84.7 to 103.9 Ω), indicating poor electrochemical stability of this WO_3_-free SSE.

**Figure 4.**
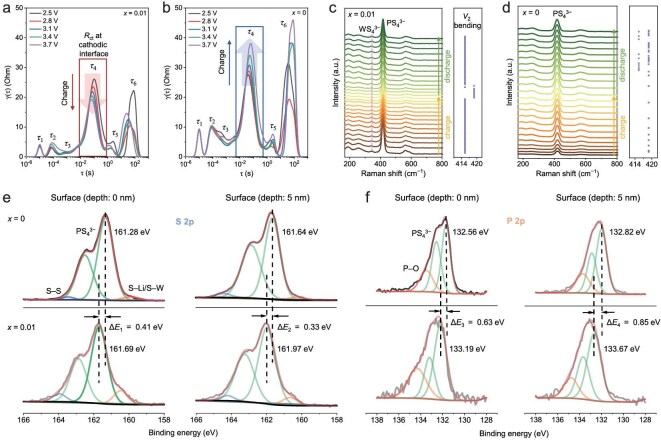
DRT plots of Li_5.5−_*_x_*P_1−_*_x_*W*_x_*S_4.5−3_*_x_*O_3_*_x_*Cl_1.5_ SSE-based full cells during the initial charging process: (a) *x* = 0.01, (b) *x* = 0. *In situ* Raman spectra at the cathodic interface of Li_5.5−_*_x_*P_1−_*_x_*W*_x_*S_4.5−3_*_x_*O_3_*_x_*Cl_1.5_ SSE-based full cells during initial charging and discharging: (c) *x* = 0.01, (d) *x* = 0. XPS spectra of Li_5.5−_*_x_*P_1−_*_x_*W*_x_*S_4.5−3_*_x_*O_3_*_x_*Cl_1.5_ SSE-based composite cathodes: (e) S 2p and (f) P 2p, measured at the 0- and 5-nm depth levels.

To decouple the impedance evolution of specific electrochemical processes during charging, DRT analysis was performed. As shown in Fig. 4a and b, six characteristic peaks were observed in the DRT plots of the cells, each representing the impedance contribution of a distinct electrochemical process to the total polarization impedance. The time constant ($\tau $) associated with each peak is indicative of the polarization process involved. Specifically, ${\tau }_1$ (∼10^−5^ s) is attributed to the grain boundary impedance of the SSE particles, ${\tau }_2$ (∼10^−4^ s) and ${\tau }_3$ (∼10^−3^ s) arise from the solid electrolyte interphase (SEI) and CEI, respectively, due to uneven charge distribution at the electrode–electrolyte interface; ${\tau }_4$ (∼10^−1^ s) and ${\tau }_5$ (∼1 s) represent the charge transfer resistance at the cathodic and anodic interfaces, respectively, while ${\tau }_6$ (∼10 s) is mainly related to Li^+^ solid-phase diffusion. Notably, the ${\tau }_4$ peak is highly correlated with the SCL effect, which is typically exacerbated at the cathodic interface during charging and thus retards Li^+^ transfer. For the full cell with optimal WO_3_-containing SSE, the ${\tau }_4$ peak intensity gradually decreases during charging, indicating that the SCL effect is largely suppressed. In contrast, the ${\tau }_4$ peak intensity increases in the DRT plots for the WO_3_-free SSE-based full cell, suggesting a more pronounced SCL effect between this SSE and CAM [50,51].

*In situ* Raman spectroscopy focused on the cathodic interface during initial cycling was employed to monitor the Li^+^ migration characteristics and the dynamic distribution of the SCL (Fig. S34). During the Li^+^ insertion and extraction process, shifts in the Raman peaks of the SSE at the cathodic interface primarily reflect stress changes and Li^+^ migration characteristics associated with volume expansion. A positive shift indicates increased stress, while a negative shift suggests a decrease. The reversible stress change in the SSE, induced by the Li-deficient SCL, is primarily attributed to internal lattice distortion. In the optimal WO_3_-containing SSE system, as shown in Fig. 4c, the characteristic peaks for both PS_4_^3−^ and WS_4_^3−^ are observable. The Raman peak for PS_4_^3−^ (${v}_2$ bending vibration, ∼415 cm^−1^) shifts to 418 cm^−1^ upon charging above 3.6 V and returns to its original position during discharge, representing a reversible stress change without altering the SSE structure. This suggests that the SCL effect is largely suppressed in this optimal SSE during cycling. In contrast, the Raman peak shift for the basic SSE system shows irregular stress changes (418 cm^−1^ ↔ 415 cm^−1^) during discharge (Fig. 4d), which is attributed to an amplified SCL effect. The Li-deficient SCL layer impedes rapid Li^+^ migration, resulting in structural damage to the SSE lattice [11]. When the stress exceeds the elastic deformation limit, irreversible plastic deformation occurs, accompanied by cracks and voids that degrade electrochemical performance. Further *in situ* Raman spectroscopy tests suggest that the SCL effect is successfully controlled in the Li_5.5−_*_x_*P_1−_*_x_*W*_x_*S_4.5−3_*_x_*O_3_*_x_*Cl_1.5_ system (*x* = 0.005) (Fig. S35), but becomes more pronounced in the system with *x* = 0.015 (Fig. S36).

Given the significant Li^+^ chemical potential difference between the SSE and CAM, Li^+^ transfers from the SSE side to the CAM side, leading to the formation of a Li-depleted SCL at the cathodic interface. This results in the creation of a reverse built-in electric field that hinders further Li^+^ transport until dynamic equilibrium is reached. The thickness of the SCL depends on the chemical state differences between the two phases and may vary as equilibrium is established. To evaluate the SCL effect between the NCM92 and Li_5.5−_*_x_*P_1−_*_x_*W*_x_*S_4.5−3_*_x_*O_3_*_x_*Cl_1.5_ SSEs (*x* = 0 and 0.01), XPS measurements were performed on the corresponding composite cathodes. The XPS energy level shifts between the composite cathodes reflect the Fermi level shift caused by the formation of the SCL [11]. As shown in Fig. 4e, f and Fig. S37, the S 2p, P 2p, and Cl 2p XPS spectra (depth of 0 and 5 nm) of the two composite cathodes were compared. In the 0-nm–depth S and P 2p XPS spectra, the PS_4_^3−^ tetrahedron peaks for the basic SSE-based composite cathode shift to lower binding energies ($\Delta {E}_1$ = 0.41 eV, $\Delta {E}_3$ = 0.63 eV) compared to the optimal SSE-based composite cathode. A similar trend was observed for Cl^−^ peaks ($\Delta {E}_5$ = 0.27 eV). The 5-nm–depth XPS spectra also show similar negative shifts for the basic SSE-based composite cathode compared to the optimal SSE system.

The observed negative shift (i.e. toward lower binding energy) in the S 2p, P 2p, and Cl 2p XPS peaks indicates a change in the local electronic environment. This shift is characteristic of an increased electron density around the S, P, and Cl anions. Within the framework of the SCL model, this is a direct signature of the Li^+^-depleted layer. The extraction of Li^+^ from the electrolyte toward the cathode (NCM92) leaves behind a localized excess of negative charge, which is inherent to the anionic framework (S, P, Cl). This anionic lattice, now in a state of effective negative charge buildup, results in the observed increase in electron density around the anions, manifesting as the XPS shift to lower binding energy. A more pronounced negative shift implies a thicker or more severe Li^+^-depleted SCL.

To elucidate why the SCL effect is significantly suppressed in the optimal WO₃-containing SSE system, DFT calculations were conducted based on the rationalized interface models shown in Fig. S38. The (110) and (100) crystal planes were selected for the CAM and SSEs at the interface, respectively. First, Bader charge analysis was performed to investigate the interfacial electronic charge transfer between the SSEs (both optimal and basic) and the CAM, with an isosurface value set at 0.002 e/Å^3^. As shown in Fig. S39, the cyan regions denote electron accumulation, while the blue regions indicate electron depletion. Although charge transfer occurs at the interface in both systems, the optimal SSE system demonstrates greater stability, with the PS_4_^3−^ tetrahedra undergoing noticeably less distortion.

**Figure 5.**
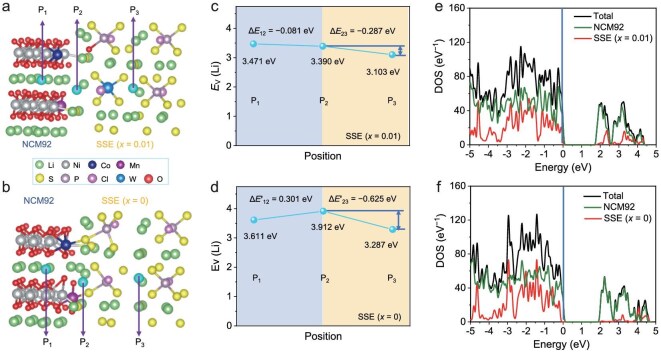
DFT calculations of the cathodic interface based on Li_5.5−_*_x_*P_1−_*_x_*W*_x_*S_4.5−3_*_x_*O_3_*_x_*Cl_1.5_ SSEs (*x* = 0.01, 0). (a, b) Optimized interfacial structures, (c, d) calculated ${E}_v$, and (e, f) PDOS. The Fermi level is indicated by the vertical dashed line in e and f.

To further examine the spatial characteristics of the SCL, the Li-vacancy formation energy (${E}_v$) at specific Li sites near the interface was calculated. Three Li sites (labeled P1, P2, and P3 in Fig. 5a and b) within the two heterojunction models were analyzed. The variations in ${E}_v$ across different sites arise from the valence band offset between the CAM and SSE. As shown in Fig. 5c, the ${E}_v$ values for the optimal SSE system show only modest fluctuations across the three positions (3.47 → 3.39 → 3.10 eV), suggesting a more uniform Li^+^ distribution at the interface. In contrast, the basic SSE system exhibits much larger variations in ${E}_v$ (3.61 → 3.91 → 3.29 eV, Fig. 5d), implying pronounced interfacial Li^+^ redistribution. Thus, incorporating optimal WO_3_ into the argyrodite SSE effectively reduces the chemical potential difference at the SSE–CAM interface, significantly weakens Li^+^ redistribution, thins the resulting SCL, and facilitates Li^+^ transport [52].

Additionally, the projected density of states (PDOS) for the two interfacial systems was analyzed. As shown in Fig. 5e and f, the valence band maximum (VBM) for both systems originates primarily from the CAM. Localized states within the band gap are also observed, attributed to crystal field splitting of the 3*d* orbitals [11]. Notably, the basic SSE system shows a higher total density of states (TDOS) at the Fermi level and more prominent electronic transport characteristics, indicating greater interfacial instability. In contrast, the optimal WO_3_-containing SSE system exhibits a lower TDOS at the Fermi level, suggesting a significant suppression of self-decomposition and structural degradation at the interface.

Summary of the logical chain: WO_3_ Substitution → Modulates SSE Fermi Level/Chemical Potential (evidenced by PDOS) → Reduces initial charge transfer and interfacial energy mismatch → Results in weaker charge redistribution (evidenced by smaller XPS binding energy shifts) → Creates a more uniform interfacial energy landscape (evidenced by flattened ${E}_v$ profile) → Facilitates easier Li^+^ migration and suppresses SCL formation.

### Application of pouch cells

**Figure 6.**
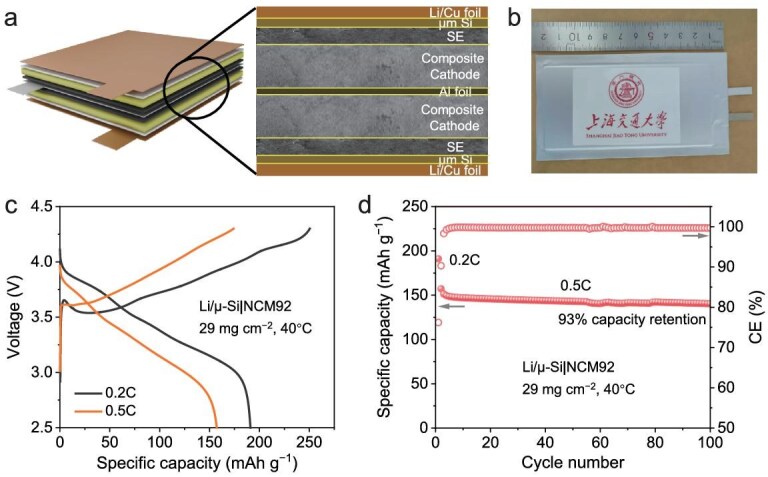
Characterization of an ASS pouch cell using the optimal WO_3_-containing SSE. (a) Schematic illustration, (b) photograph of the assembled cell, (c) charge-discharge curves at 0.2C and 0.5C, (d) cycling performance at 0.5C.

To assess the practical applicability of the optimal WO_3_-containing SSE in ASSLBs, a high-CAM-loading (29 mg cm^−2^) ASS pouch cell was assembled and tested. The cell featured a double-layer configuration, with each layer measuring 10 cm × 5 cm (Fig. 6a and b). A Li/*μ*-Si film anode was used to facilitate cell assembly. Testing was conducted at 40°C under a pressure of 50 MPa. Figure 6c shows the charge–discharge profiles at 0.2C and 0.5C. At 0.2C, the cell delivers an initial specific capacity of 191 mAh g^−1^ with an initial CE of 76%, corresponding to a total capacity of 554 mAh. Even at the higher rate of 0.5C, the cell maintains a high specific capacity of 157 mAh g^−1^. Based on the total mass of the cathode, electrolyte, and anode (Table S11), the gravimetric energy density of the pouch cell at 0.2C was calculated to be 363 Wh kg^−1^. The cycling performance of the cell is presented in Fig. 6d. After 100 cycles at 0.5C, the cell retains 93% of its capacity, indicating excellent cycling stability. These results collectively demonstrate that the modified argyrodite SSE is a highly promising electrolyte for practical ASSLB applications.

## CONCLUSIONS

In summary, we have successfully introduced appropriate WO_3_ into argyrodite to develop an optimal SSE with significantly enhanced electrochemical properties, such as the highest reported ionic conductivity (13.5 mS cm^−1^ at 25°C) among O-substituted argyrodite SSEs. As a result, full cells employing this SSE and NCM92 CAM demonstrate outstanding performance, delivering a high specific capacity of 217 mAh g^−1^ at 0.1C and exceptional long-term cycling stability, with 92% and 80% capacity retention after 1000 and 5000 cycles at 1C and 5C, respectively. This performance markedly surpasses that of the cell employing the unsubstituted baseline SSE, which achieves only 200 mAh g^−1^ at 0.1C and fails after just 408 cycles at 1C.

Further experimental characterizations and DFT calculations reveal that the superior electrochemical performance arises not only from the high ionic conductivity and interfacial stability of the optimal SSE, but also from the ability of WO_3_ substitution to tune the chemical potential of the SSE. This adjustment brings it closer to that of the CAM, thereby effectively suppressing the SCL effect. Our work offers a new perspective on mitigating the SCL effect in ASSLBs—by tailoring the chemical potential of the SSE to reduce the mismatch with the CAM—paving the way for more stable and high-performance ASSLBs.

## METHODS

Detailed materials and methods are available in the online Supplementary data.

## Supplementary Material

nwag015_Supplemental_File
