# Supplementary material for: Space charge regulation for ultra-stable all-solid-state lithium batteries by engineering of argyrodite electrolyte
Source: Natl Sci Rev. 2026 Jan 10;13(5):nwag015. doi: 10.1093/nsr/nwag015 (PMC12988355; doi:10.1093/nsr/nwag015)
Supplement: nwag015_Supplemental_File [file nwag015_supplemental_file.pdf]

Supporting information

Space charge regulation for ultra-stable all-solid-state lithium batteries by engineering of argyrodite electrolyte

Jingjing Wang[1,+], Linan Jia[2,+], Yibo Du[2], Bangjun Guo[2], Haozhe Geng[1], Qianjin Huang[1], Junbo Hou[3], Jinhui Zhu[1,]* and Xiaodong Zhuang[1,4,]*

[1]The Soft2D Lab, State Key Laboratory of Synergistic Chem-Bio Synthesis, State Key Laboratory of Metal Matrix Composites, Shanghai Key Laboratory of Electrical Insulation and Thermal Ageing, School of Chemistry and Chemical Engineering, Shanghai Jiao Tong University, Shanghai 200240, China

E-mail: zhujinhui1109@sjtu.edu.cn (J. Zhu); zhuang@sjtu.edu.cn (X. Zhuang)

[2]School of Mechanical Engineering, Shanghai Jiao Tong University, Shanghai 200240, China

[3]Power System Resources Environmental Technology Co., Ltd., 585 Changan North Road, Jiaxing 314399, China

[4]Frontiers Science Center for Transformative Molecules, Zhanjiang Institute for Advanced Study, Shanghai Jiao Tong University, Shanghai 201203, China

[+] These authors contributed equally to this work

Materials synthesis

Synthesis of $Li_{5.5}PS_{4.5}Cl_{1.5}$ SSE

The $Li_{5.5}PS_{4.5}Cl_{1.5}$ SSE was synthesized via a conventional solid-state reaction method following previously reported procedures [1, 2]. In a typical synthesis, stoichiometric amounts of high-purity precursors—Li_2S (Alfa Aesar, 99.9%), P_2S_5 (Macklin, 99%), and LiCl (Sigma-Aldrich, 99.9%)—were thoroughly mixed in a zirconia vial and subjected to high-energy ball milling at 550 rpm for 15 h. The resulting powder was then pelletized under pressure and sealed in quartz tubes under argon atmosphere. The samples were subsequently annealed at 480 °C for 6 h with a controlled heating rate of 0.5 °C min^{-1}. After sintering, the pellets were manually ground into powders using an agate mortar. All handling procedures were performed in an argon-filled glove box (H_2O < 0.1 ppm, O_2 < 0.1 ppm) to prevent moisture and oxygen contamination.

Synthesis of $Li_{5.5-x}P_{1-x}W_xS_{4.5-3x}O_{3x}Cl_{1.5}$ SSEs (x = 0.005, 0.01, 0.015)

The WO_3-substituted SSEs were prepared using a similar solid-state synthesis protocol. Appropriate molar ratios of Li_2S (Alfa Aesar, 99.9%), P_2S_5 (Macklin, 99%), LiCl (Adamas, 99.99%), and WO_3 (Adamas, 99.9%) were employed as starting materials. The synthesis procedure followed the same ball milling, pelletization, and thermal treatment conditions as described for the WO_3-free SSE.

Synthesis of refined $Li_{5.5-x}P_{1-x}W_xS_{4.5-3x}O_{3x}Cl_{1.5}$ SSEs (x = 0, 0.005, 0.01, 0.015)

The as-prepared ground SSE powder was refined via wet ball milling. Specifically, a mixture of the ground SSE powder and anhydrous diethyl ether (in a 1:2 weight ratio) was ball-milled at 400 rpm for 10 h. The solvent was subsequently removed by centrifugation, and the resulting precipitate was vacuum-dried at 60 °C to obtain the refined SSEs.

Synthesis of composite cathodes

Composite cathodes were fabricated by mechanochemical mixing through ball milling. The cathode formulation consisted of: $LiNi_{0.92}Co_{0.05}Mn_{0.03}O_2$ (NCM92, MINMETALS NEW ENERGY MATERIALS (HUNAN) CO., LTD) as active material (70 wt%); as-synthesized refined SSE powder as ionic conductor (29 wt%); vapor-grown carbon fiber (VGCF, Canrd Technology Co., Ltd.) as conductive additive (1 wt%). The components were mixed by ball milling at 400 rpm for 8 h under continuous argon flow to ensure homogeneous distribution while preventing atmospheric contamination.

Materials characterization

The morphology and elemental composition of the synthesized SSEs were characterized using a scanning electron microscope (SEM, EmCrafts CUBE II) equipped with energy-dispersive spectroscopy (EDS). For detailed microstructural analysis, transmission electron microscopy (TEM, FEI Tecnai G2 F20) and high-resolution TEM (HRTEM) were employed. Additionally, scanning transmission electron microscopy–energy-dispersive X-ray spectroscopy (STEM-EDS) was performed on the same instrument to examine the elemental distribution at the nanoscale. The crystal structure of the SSEs was analyzed via X-ray diffraction (XRD, D8 ADVANCE Da Vinci, Cu Kα radiation, λ = 1.5406 Å) over a 2θ range of 10° to 80°. The obtained XRD patterns were refined using the Rietveld method implemented in the General Structure Analysis System (GSAS II) software to extract lattice parameters. Raman spectroscopy (K-Sens-532, 532 nm excitation laser) was used to probe local bonding environments and phase purity. The surface chemical composition was investigated by X-ray photoelectron spectroscopy (XPS, AXIS UltraDLD), providing insights into oxidation states and possible surface contaminants. Furthermore, solid-state ^{7}Li magic-angle spinning nuclear magnetic resonance (MAS NMR) spectra were acquired on a Bruker AVANCE III 400 (9.4 T) spectrometer equipped with a dual-resonance CP/MAS probe operating at 155.5 MHz. The ^{7}Li

MAS NMR experiments were performed using a radiofrequency field strength of 143 kHz, a 0.9 μs 45° pulse, a relaxation delay of 0.5 s, and 800 accumulated scans for sufficient signal-to-noise ratio. The ^{7}Li chemical shifts were referenced to a 1 M LiCl aqueous solution (0 ppm). Finally, the crystal structure visualization was performed using VESTA software for atomic-scale modeling and representation.

Electrochemical characterizations

Ionic conductivity (σ_i) measurement

The σ_i of the as-prepared SSEs was evaluated via electrochemical impedance spectroscopy (EIS) using an EC-Lab SP-300 workstation. Measurements were conducted across a temperature range of 25–70 °C with an applied AC amplitude of 10 mV over a frequency range of 0.1 Hz to 7.0 MHz.

For testing, 120 mg of SSE powder was uniaxially pressed into pellets (10 mm diameter, ~1 mm thickness) under 350 MPa in a PEEK die, then sandwiched between two stainless steel (SS) blocking electrodes. The σ_i was calculated using:

$$\sigma_i = \frac{l}{R \times s} \# Eq. 1$$

where l, s, and R denote the sample thickness, effective area of the electrode, and impedance of the sample, respectively.

Electronic conductivity (σ_e) measurement

The σ_e was determined using DC polarization in an SS|SSE|SS symmetric cell. A stepwise voltage application (0.2, 0.4, 0.6, and 0.8 V) was employed, and the steady-state current was recorded to calculate σ_e via Ohm's law.

Electrochemical stability window (ESW) assessment

The ESW was probed by cyclic voltammetry (CV) at 0.1 mV s^{-1} (0–5.0 V vs. Li/Li^{+}) using a CHI660B workstation. The cell configuration comprised: Working electrode: A composite of SSE + carbon black (70:30 wt%) (10 mg), cold-pressed at 300 MPa;

and counter/reference electrode: Lithium metal foil.

Assembly and measurements of cells

Symmetric Li|Li cells

Symmetric cells were fabricated by pressing 100 mg of as-prepared ground SSE powder into a pellet under 300 MPa, and then Li metal pieces were attached to both sides of the pellet under 15 MPa using a Swagelok cell assembly. All procedures were conducted in an argon-filled glovebox with H_2O and O_2 levels below 1 ppm. The critical current densities (CCDs) of the symmetric cells were evaluated. The initial current density was set at 0.1 mA cm^{-2} and incrementally increased by 0.1 mA cm^{-2} per step. Each charge–discharge cycle lasted 1 h. A sharp voltage drop was considered the onset of a short circuit, and the corresponding current density was defined as the CCD. Long-term cycling stability tests were conducted at current densities of 0.1 mA cm^{-2} and 0.5 mA cm^{-2}.

Li–In|NCM92 full cells

All assembly procedures were performed in an argon-filled glovebox (H_2O < 0.1 ppm, O_2 < 0.1 ppm). Cells were constructed in a polyether ether ketone (PEEK) die with a diameter of 10 mm. First, 100 mg of as-prepared ground SSE powder was pelletized under 150 MPa for 1 min. Then, 10 mg of the composite cathode was evenly distributed over one side of the pellet and pressed at 320 MPa for 1 min. A Li–In foil was attached to the opposite side. Al and Cu foils were applied to the cathode and anode sides, respectively, as current collectors. Prior to testing, the assembled cell mold was subjected to 35 MPa of stack pressure using a digital tablet press, and the cell was secured between two steel plates with bolts at each corner to maintain pressure.

Galvanostatic charge–discharge cycling was performed at 25 °C using a NEWARE Battery Testing System (CT-4008Tn-5V20mA-HWX). Cycling tests were conducted within a voltage range of 2.5–3.7 V. Rate capability was evaluated at current rates

ranging from 0.1C to 5C (1C = 200 mA g^{-1}), with five cycles per rate. For long-term cycling tests, the initial two cycles were run at 0.1C, followed by continuous cycling at 1C or 2C or 5C.

For galvanostatic intermittent titration technique (GITT) measurements, the Li^+ diffusion coefficient (D_{Li^+}) was calculated using Equation 2:

$$D_{Li^+} = \frac{4}{\pi\tau}\left(\frac{m_B V_M}{M_B S}\right)^2 (\frac{\Delta E_s}{\Delta E_t})^2 \qquad Eq. 2$$

In this equation, τ is the duration of the constant current pulse; m_B, V_M, M_B, and S represent the active material mass (g), molar volume (cm^3 mol^{-1}), molar mass (g mol^{-1}), and electrode–electrolyte interface area (cm^2), respectively. ΔE_s and ΔE_t refer to the steady-state voltage change and the total voltage change during the pulse, respectively. Cells were first charged at 0.1C to 3.7 V, then discharged for 10 min at 0.1C, followed by a 60-min rest period until the voltage reached 2.5 V.

EIS measurements were performed using a Bio-Logic SP300 potentiostat over a frequency range of 100 kHz to 0.1 Hz with a 10-mV amplitude.

Li|NCM92 full cells

The Li|NCM92 full cells were assembled following a procedure similar to that used for the Li–In|NCM92 full cells, with the Li–In anode replaced by Li metal anode (LMA). A testing pressure of 15 MPa was applied.

Li/μSi|NCM92 pouch cell

The composite cathode and SE membranes were prepared via a dry process using polytetrafluoroethylene (PTFE) as a binder. The cathode composition was 70% NCM92, 28% optimal SSE (refined), 1% VGCF, and 1% PTFE. The SE membrane prepared using ground optimal SSE powder including 0.5% PTFE binder. The Li/μ-Si anode membrane was fabricated by coating a slurry [96% μ-Si (Canrd Technology Co., Ltd.) and 4% polyisobutene in ultradry n-hexane] onto a 20 μm-thick Li foil, followed by vacuum drying.

The pouch cell was assembled by sequentially stacking: Cu foil, Li/μ-Si anode

membrane, SSE membrane, composite cathode membrane, Al foil, another composite cathode membrane, another SSE membrane, a second Li/μ-Si anode membrane, and a final Cu foil. This stack was sealed in an Al-plastic pouch. The cell was clamped between iron plates and tightened with screws under 50 MPa for cycling tests.

Electrochemical performance was evaluated using a NEWARE Battery Testing System (CT-4008-5V12A-DB) at 40 °C, with a voltage window of 2.5–4.3 V. The cell was cycled at 0.2C for the initial cycle and at 0.5C for subsequent cycles.

Theoretical calculations

All density functional theory (DFT) calculations were performed using the Vienna Ab Initio Simulation Package (VASP), within the framework of the generalized gradient approximation (GGA) as formulated by Perdew, Burke, and Ernzerhof (PBE). The projector augmented wave (PAW) method was employed to represent the ionic cores, and a plane-wave basis set with a kinetic energy cutoff of 550 eV was used to describe the valence electrons. Partial occupancies of the Kohn–Sham orbitals were handled using the Gaussian smearing method with a smearing width of 0.1 eV. The electronic energy was considered converged when the total energy change was less than 10^{-5} eV. Geometry optimization was deemed complete when the forces on all atoms were below 0.05 eV/Å. To account for the strong on-site Coulomb interactions of the Ni, Co, and Mn 3d electrons, a Hubbard U correction (DFT+U) was applied. The U values, taken from literature, were set as 6.88 eV for Ni, 5.95 eV for Co, and 5.2 eV for Mn. Grimme's DFT-D3 dispersion correction scheme was incorporated to account for van der Waals interactions. Brillouin zone integrations were carried out using a Monkhorst-Pack k-point mesh of 3×3×2. The Li-vacancy formation energy (E_f) was calculated using the Equation 3:

$$E_f = E_v + E_{Li} - E_{Pure} \# Eq.3$$

where E_v and E_{pure} are the total energies of the relaxed structures with and without a Li vacancy, respectively, as obtained from DFT+U calculations. E_{Li} represents the chemical potential of Li, which was set equal to the energy per atom of bulk Li metal.

Supplementary Figures

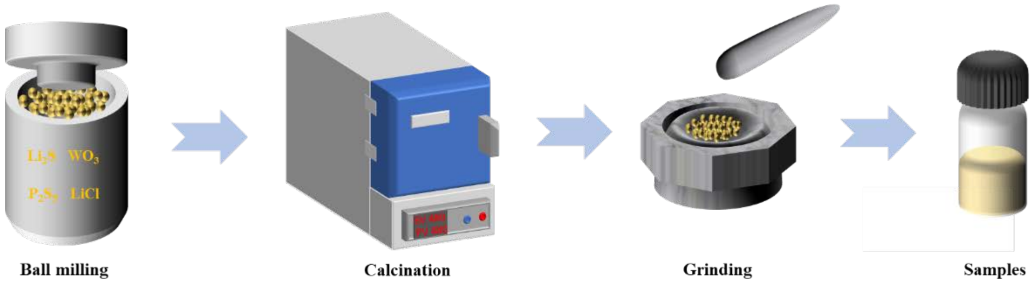

Figure S1 Synthesis of $Li_{5.5-x}P_{1-x}W_xS_{4.5-3x}O_{3x}Cl_{1.5}$ SSEs (x = 0, 0.005, 0.01, 0.015).

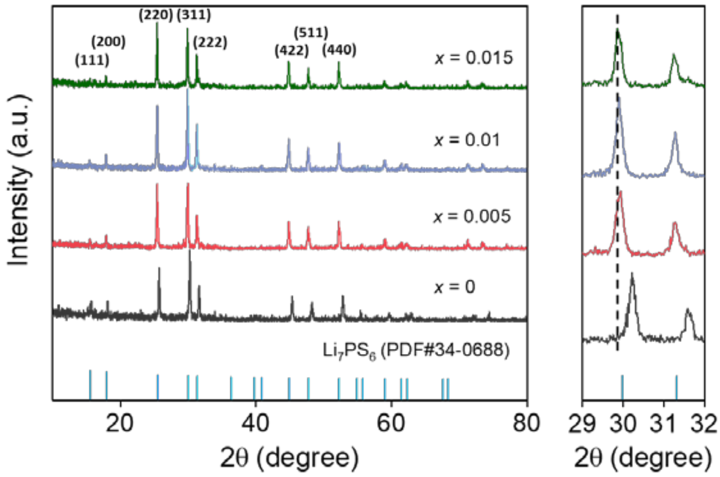

Figure S2 The XRD patterns of $Li_{5.5-x}P_{1-x}W_xS_{4.5-3x}O_{3x}Cl_{1.5}$ SSEs (x = 0, 0.005, 0.01, 0.015).

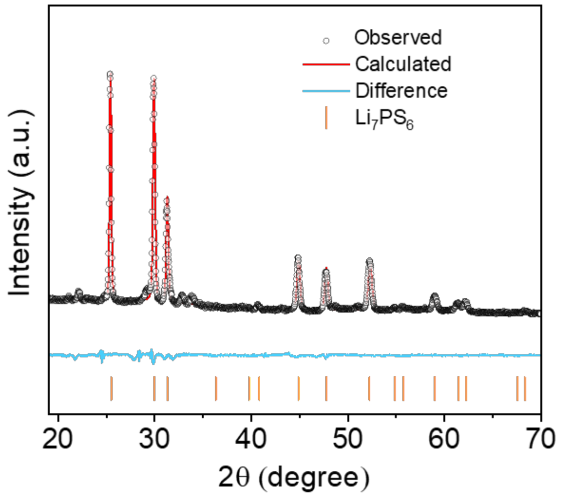

Figure S3 XRD Rietveld refinement spectra of $Li_{5.5}PS_{4.5}Cl_{1.5}$ SSE.

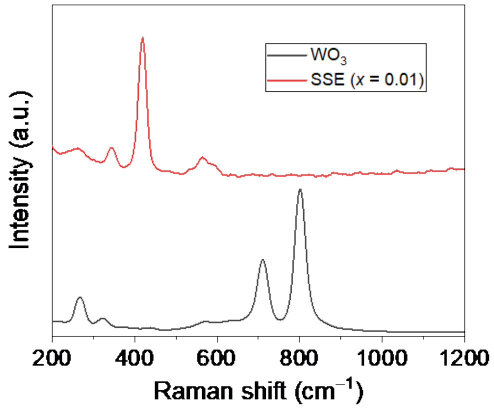

Figure S4 Raman spectra of WO_3 and synthesized SSE (x = 0.01).

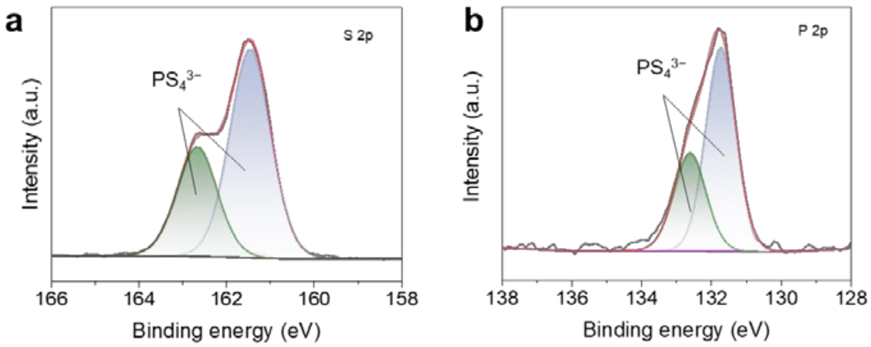

Figure S5 S 2p (a) and P 2p (b) XPS spectra of $Li_{5.5}PS_{4.5}Cl_{1.5}$ SSE.

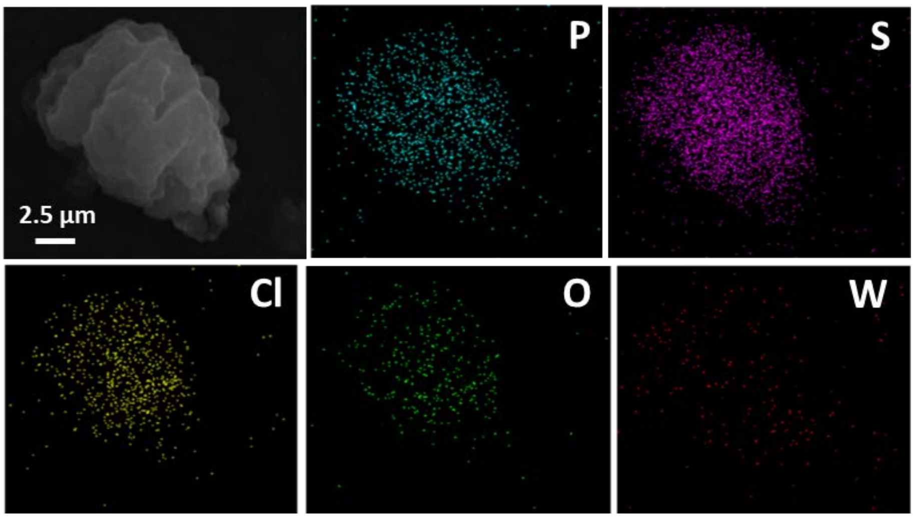

Figure S6 The SEM image and mappings of ground $Li_{5.49}P_{0.99}W_{0.01}S_{4.47}O_{0.03}Cl_{1.5}$ SSE powder.

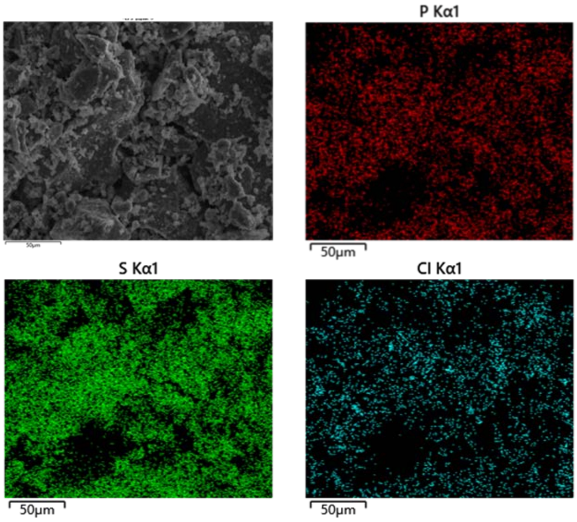

Figure S7 The SEM image and mappings of ground $Li_{5.5}PS_{4.5}Cl_{1.5}$ SSE powder.

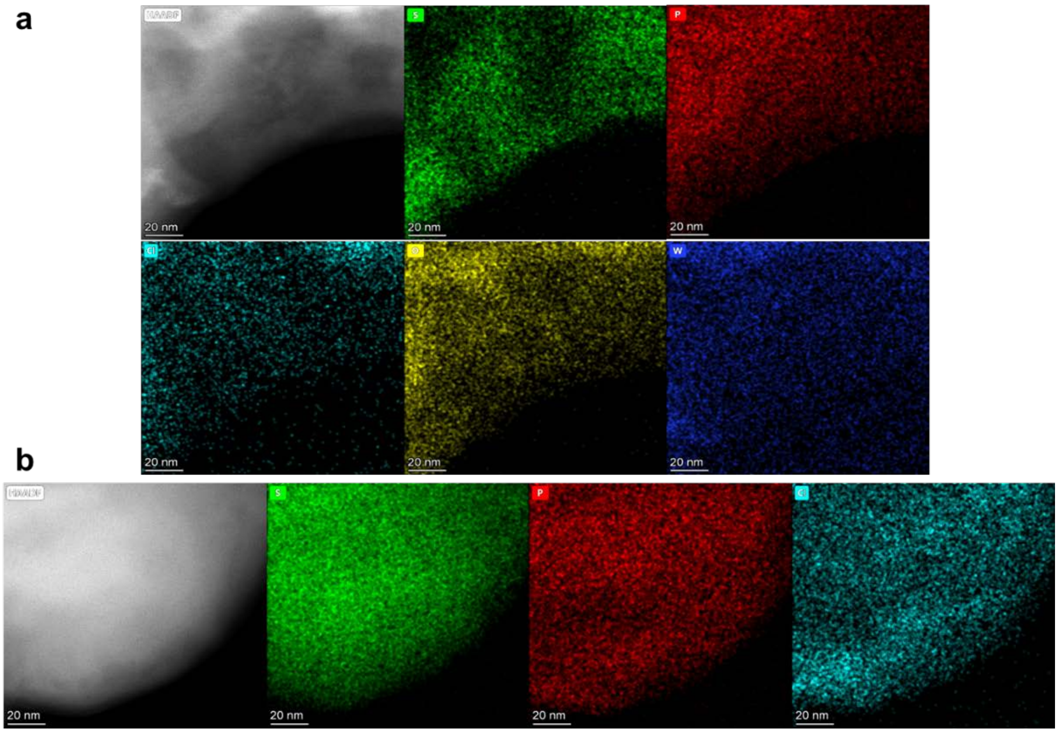

Figure S8 HAADF-STEM image and corresponding EDS mappings of ground $Li_{5.49}P_{0.99}W_{0.01}S_{4.47}O_{0.03}Cl_{1.5}$ SSE (a), and $Li_{5.5}PS_{4.5}Cl_{1.5}$ SSE (b).

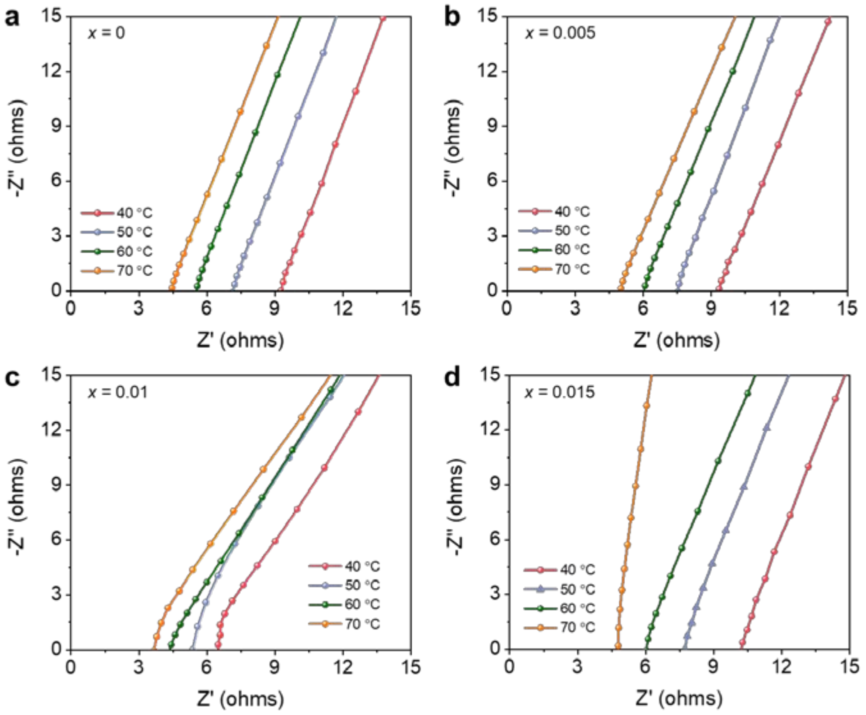

Figure S9 Nyquist plots of $Li_{5.5-x}P_{1-x}W_xS_{4.5-3x}O_{3x}Cl_{1.5}$ SSEs (x = 0, 0.005, 0.01, 0.015) in the temperature range of 40 ℃ to 70 ℃.

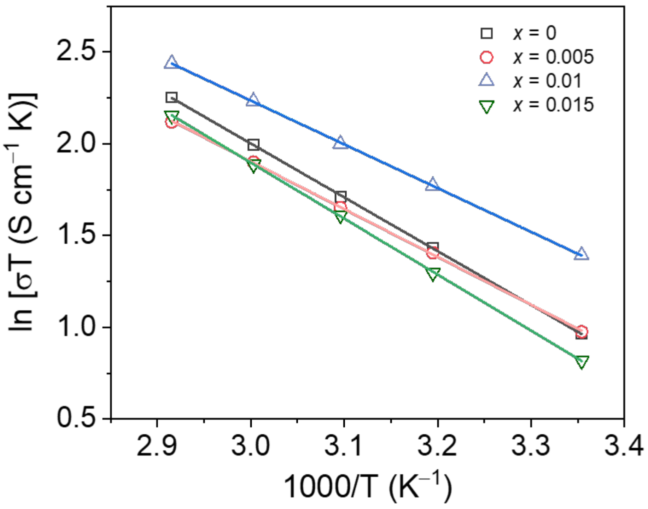

Figure S10 Arrhenius curves of $Li_{5.5-x}P_{1-x}W_xS_{4.5-3x}O_{3x}Cl_{1.5}$ SSEs (x = 0, 0.005, 0.01, 0.015).

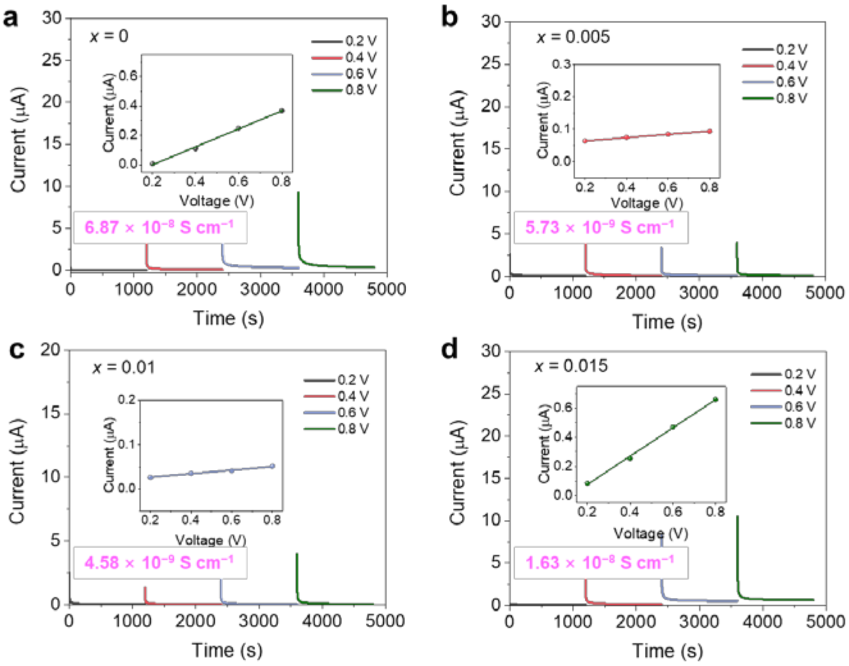

Figure S11 Stable current response of $Li_{5.5-x}P_{1-x}W_xS_{4.5-3x}O_{3x}Cl_{1.5}$ SSEs (x = 0, 0.005, 0.01, 0.015) at different voltage.

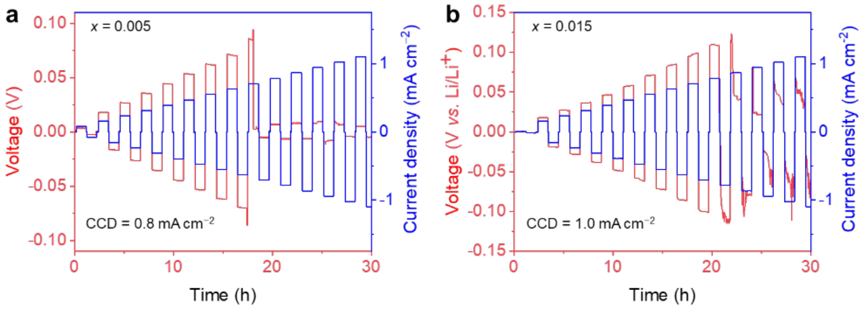

Figure S12 CCD tests of Li|Li cells with $Li_{5.5-x}P_{1-x}W_xS_{4.5-3x}O_{3x}Cl_{1.5}$ SSEs. (a) x = 0.005. (b) x = 0.015.

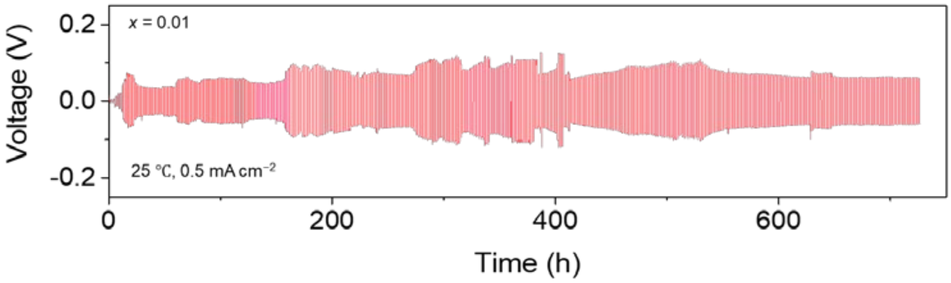

Figure S13 Voltage profile of Li|Li cell with $Li_{5.49}P_{0.99}W_{0.01}S_{4.47}O_{0.03}Cl_{1.5}$ SSE.

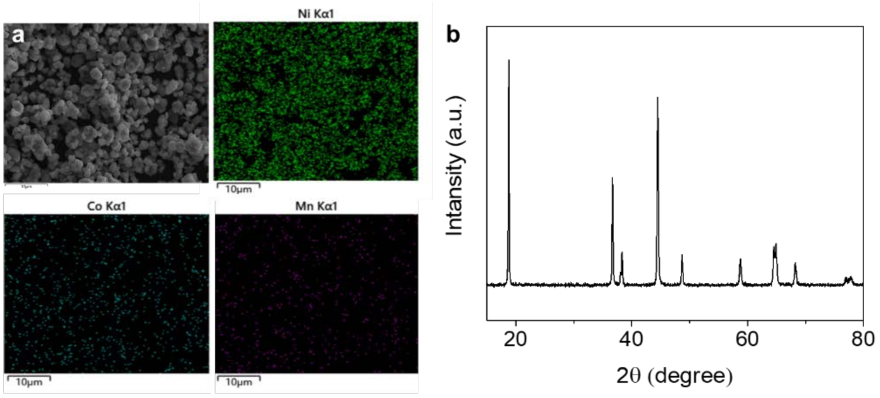

Figure S14 SEM image and mappings (a), and XRD pattern (b) of NCM92 CAM.

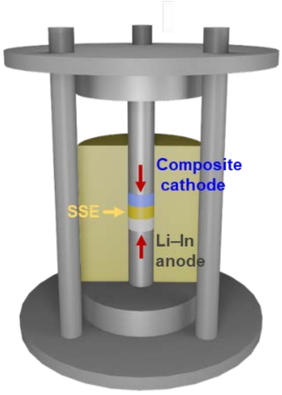

Figure S15 Schematic diagram of Li–In|SSE|NCM92 full cell.

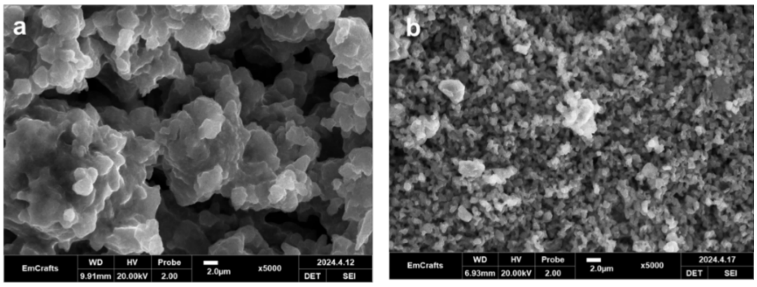

Figure S16 SEM images of ground (a) and refined (b) $Li_{5.49}P_{0.99}W_{0.01}S_{4.47}O_{0.03}Cl_{1.5}$ SSE.

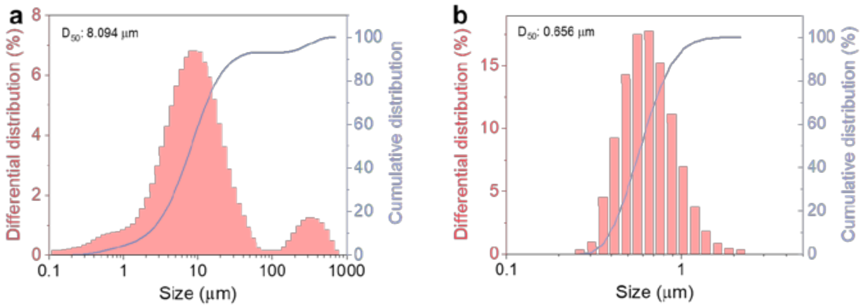

Figure S17 The particle size distribution of ground (a) and refined (b) $Li_{5.49}P_{0.99}W_{0.01}S_{4.47}O_{0.03}Cl_{1.5}$ SSE.

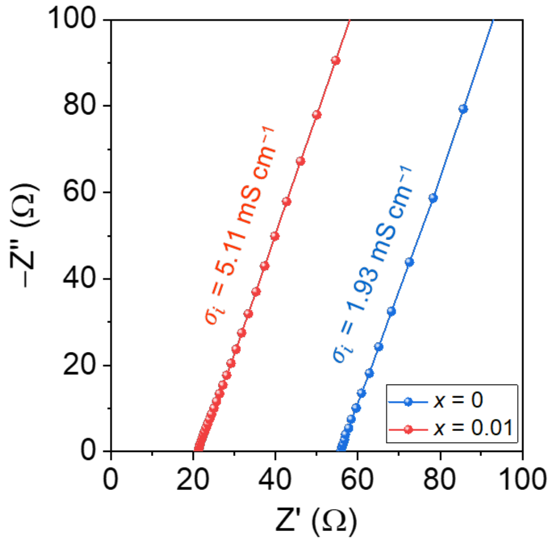

Figure S18 σ_i of refined SSEs.

The ionic conductivity (σ_i) of the refined optimal and basic SSEs was measured and calculated to be 5.11 and 1.93 mS cm^{-1}, respectively. These values further support the enhanced performance observed with the optimal SSE.

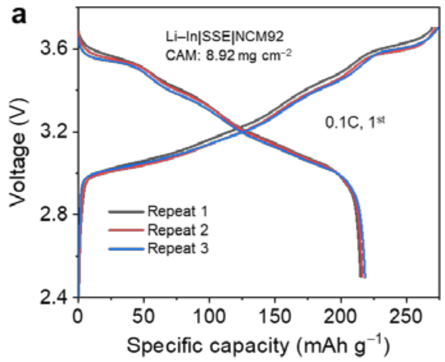

b

	Specific capacity (mAh g^{-1})	CE (%)
Repeat 1	215.1	79.8
Repeat 2	216.6	79.7
Repeat 3	218.4	79.8
Average value	216.7	79.77
Standard deviation	1.65	0.058

Figure S19 (a) Repeat tests of full cells with $Li_{5.49}P_{0.99}W_{0.01}S_{4.47}O_{0.03}Cl_{1.5}$ SSE. (b) The calculated average specific capacity and CE, and the resulted standard deviation.

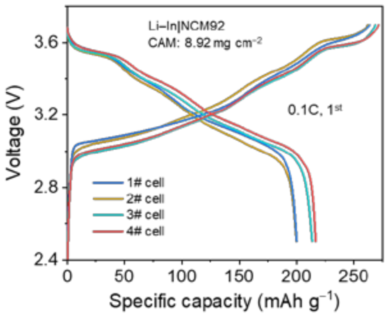

Full cells	Specific capacity (mAh g^{-1})	CE (%)
1#: Li–In|SSE(x=0)|NCM92/SSE(x=0)	200	75.9
2#: Li–In|SSE(x=0.01)|NCM92/SSE(x=0)	200	76.2
3#: Li–In|SSE(x=0)|NCM92/SSE(x=0.01)	214	79.3
4#: Li–In|SSE(x=0.01)|NCM92/SSE(x=0.01)	217	79.7

Note: The SSE for the separator was used in its ground form, whereas the catholyte was the refined SSE.

Figure S20 Specific capacities of full cells with different configurations.

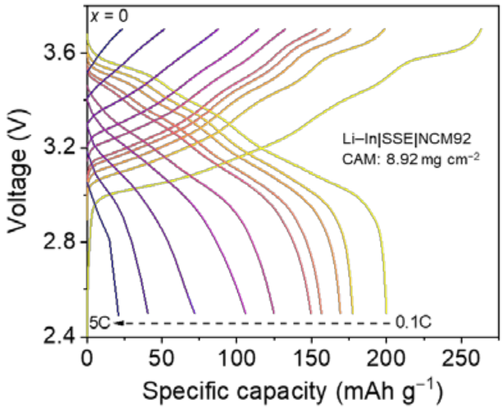

Figure S21 Voltage profiles of $Li_{5.5}PS_{4.5}Cl_{1.5}$ SSE-based full cell under various rates.

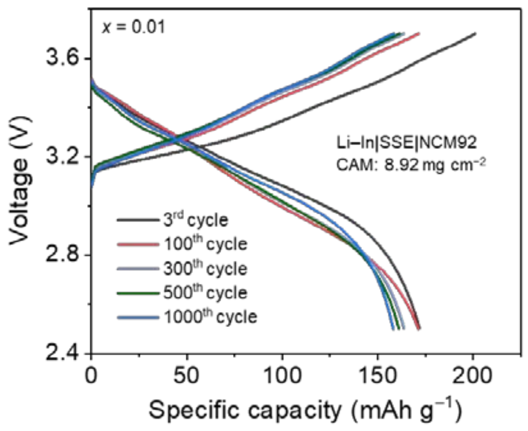

Figure S22 Voltage profiles of $Li_{5.49}P_{0.99}W_{0.01}S_{4.47}O_{0.03}Cl_{1.5}$ SSE-based full cell under 1C at different cycle numbers.

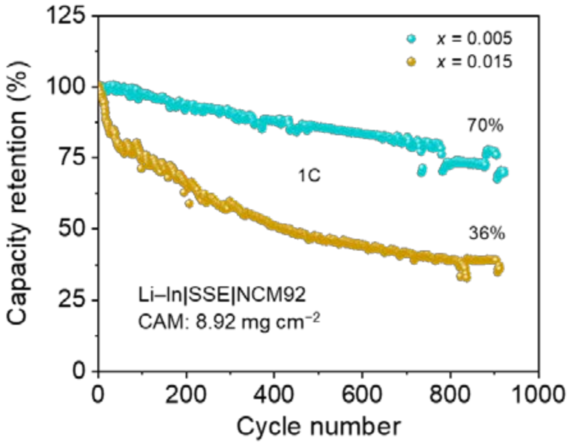

Figure S23 Cycling performance of the full cells with $Li_{5.5-x}P_{1-x}W_xS_{4.5-3x}O_{3x}Cl_{1.5}$ SSEs (x = 0.005, 0.015).

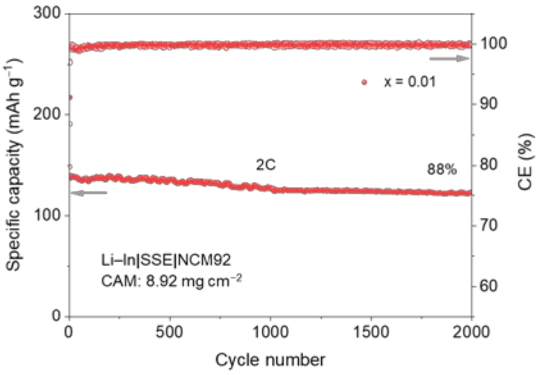

Figure S24 Cycling performance of $Li_{5.49}P_{0.99}W_{0.01}S_{4.47}O_{0.03}Cl_{1.5}$ SSE-based full cell at 2C.

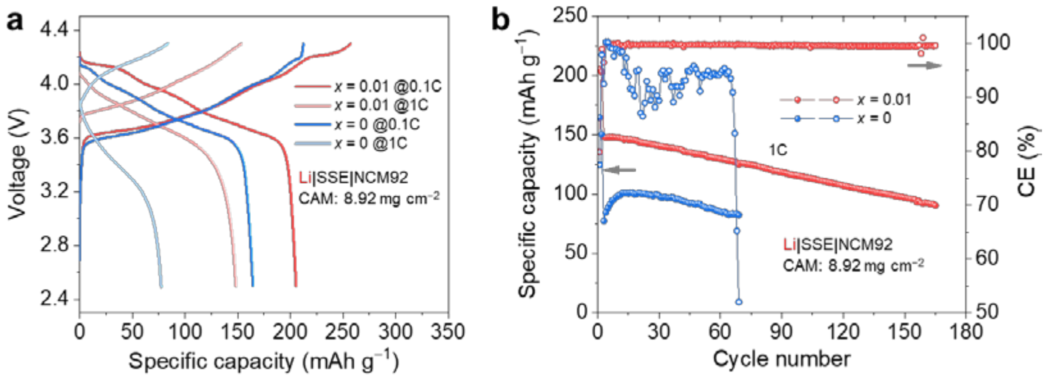

Figure S25 Voltage profiles (a) and cycling stability (b) of Li|NCM92 full cells using $Li_{5.5\text{-}x}P_{1\text{-}x}W_xS_{4.5\text{-}3x}O_{3x}Cl_{1.5}$ SSEs (x = 0.01, 0).

The full cells employing the optimal and basic SSEs delivered specific capacities of 205 and 165 mAh g^{-1} at 0.1C, with initial CEs of 80% and 77%, respectively. Further cycling tests at 1C revealed that the cell with the optimal SSE maintained stable operation for 165 cycles, retaining 61% of its capacity. In contrast, the cell using the basic SSE exhibited lower and fluctuating CEs (averaging 93%) and failed after 69 cycles. These results confirm the superior stability of the optimal SSE over the basic SSE when paired with a LMA.

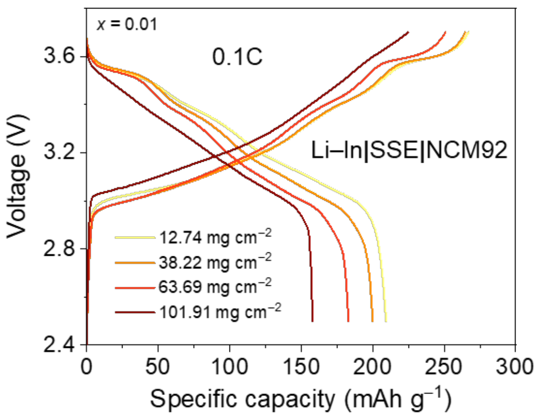

Figure S26 Voltage profiles of $Li_{5.49}P_{0.99}W_{0.01}S_{4.47}O_{0.03}Cl_{1.5}$ SSE-based full cell under 0.1C with various CAM mass loading.

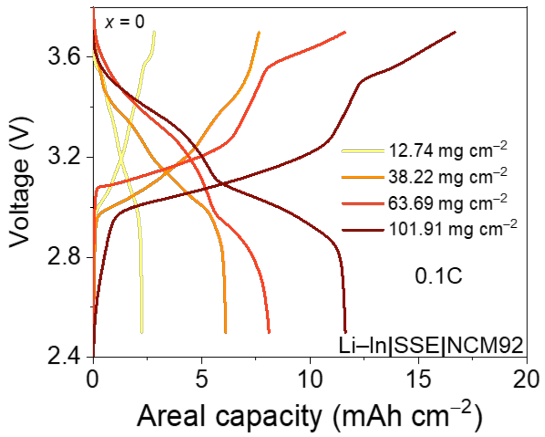

Figure S27 Voltage profiles of $Li_{5.5}PS_{4.5}Cl_{1.5}$ SSE-based full cell under 0.1C with various CAM mass loading.

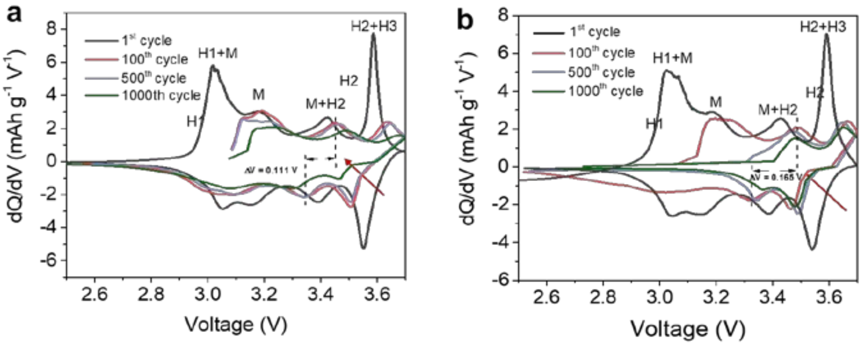

Figure S28 dQ/dV curves of full cells with $Li_{5.5\text{-}x}P_{1\text{-}x}W_xS_{4.5\text{-}3x}O_{3x}Cl_{1.5}$ SSEs. (a) x = 0.01, (b) x = 0.

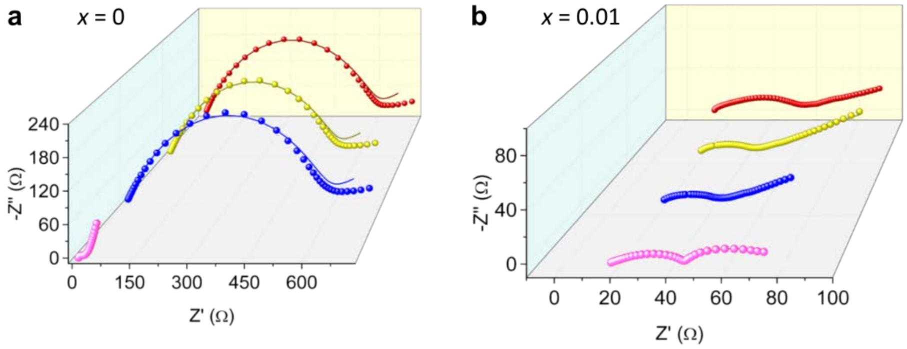

Figure 29 EIS spectra of $Li_{5.5-x}P_{1-x}W_xS_{4.5-3x}O_{3x}Cl_{1.5}$ SSE-based full cells after various cycling. (a) $x = 0$, (b) $x = 0.01$. Pink: 0 cycle; bule: 300 cycles; yellow: 500 cycles; red: 1000 cycles.

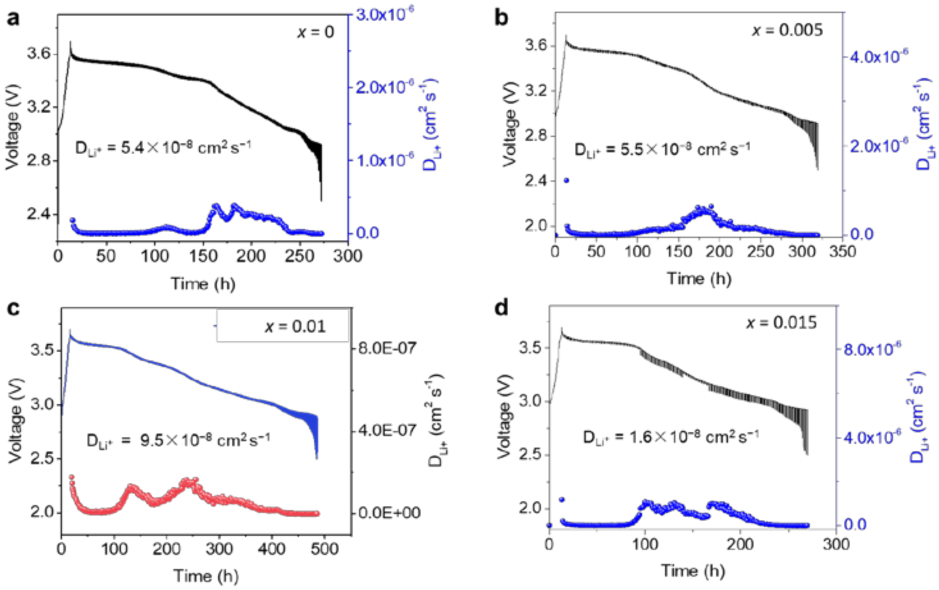

Figure S30 GITT plots and the corresponding D_{Li^+} curves of the full cells with $Li_{5.5-x}P_{1-x}W_xS_{4.5-3x}O_{3x}Cl_{1.5}$ SSEs. (a) x = 0, (b) x = 0.005, (c) x = 0.01, and (d) x = 0.015.

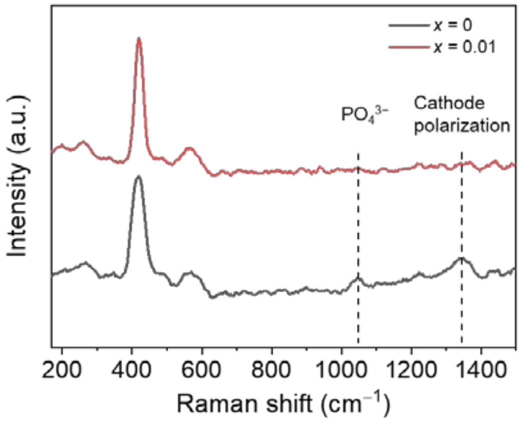

Figure S31 Raman spectra of the composite cathodes with $Li_{5.5-x}P_{1-x}W_xS_{4.5-3x}O_{3x}Cl_{1.5}$ SSEs (x = 0, 0.01) after 100 cycles.

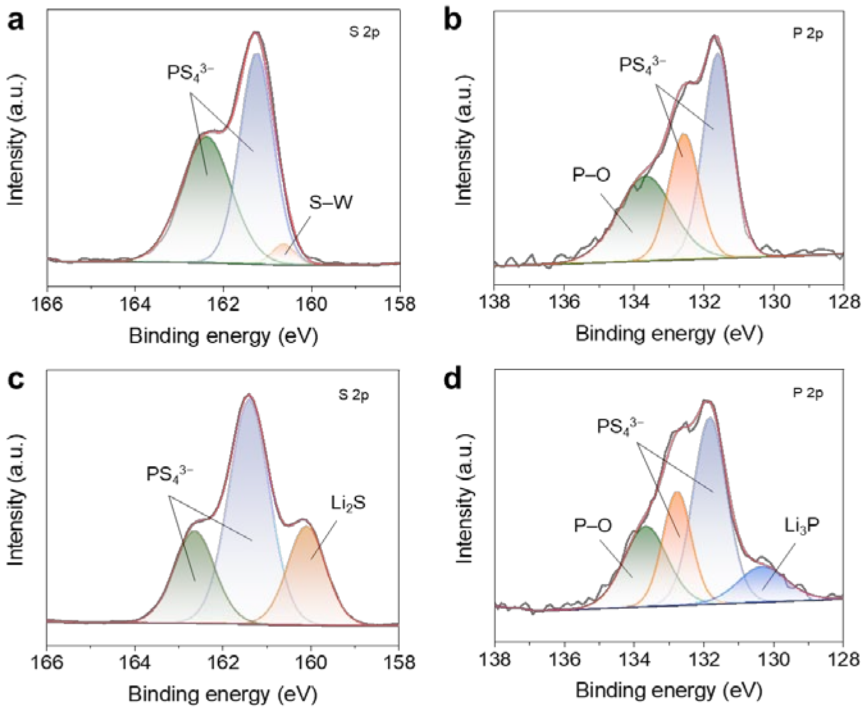

Figure S32 S 2p and P 2p XPS spectra of $Li_{5.49}P_{0.99}W_{0.01}S_{4.47}O_{0.03}Cl_{1.5}$ SSE (a, b) and $Li_{5.5}PS_{4.5}Cl_{1.5}$ SSE (c, d) after 100 cycles, respectively.

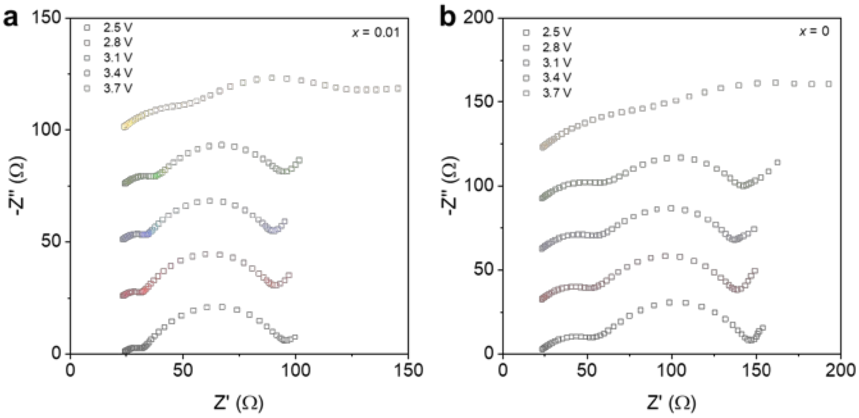

Figure S33 Nyquist plots of full cells with $Li_{5.5-x}P_{1-x}W_xS_{4.5-3x}O_{3x}Cl_{1.5}$ SSEs during initial charging. (a) x = 0.01, (b) x = 0.

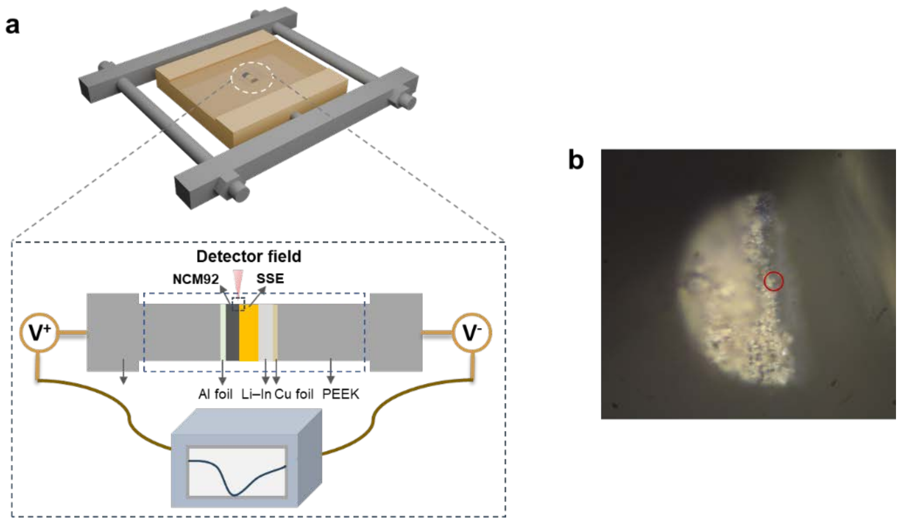

Figure S34 (a) Schematic diagram of set up for in situ Raman spectroscopy tests. (b) Optical images of laser beam focused area during the *in situ* Raman spectroscopy tests.

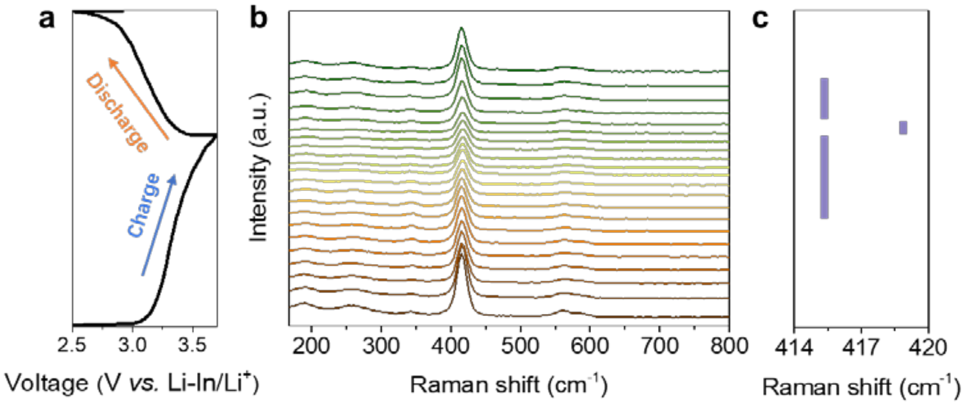

Figure S35 In situ Raman spectra of cathodic interface of $Li_{5.5-x}P_{1-x}W_xS_{4.5-3x}O_{3x}Cl_{1.5}$ SSE-based full cell during initial charging and discharging, where x = 0.005.

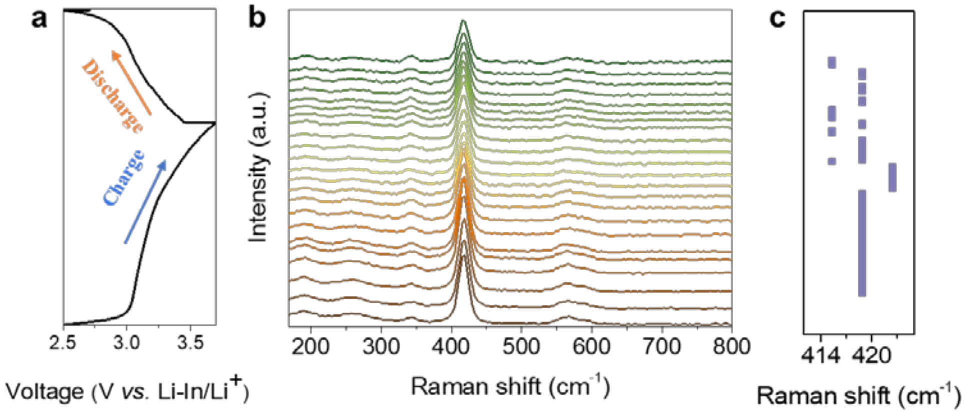

Figure S36 In situ Raman spectra of cathodic interface of $Li_{5.5-x}P_{1-x}W_xS_{4.5-3x}O_{3x}Cl_{1.5}$ SSE-based full cell during initial charging and discharging, where $x = 0.015$.

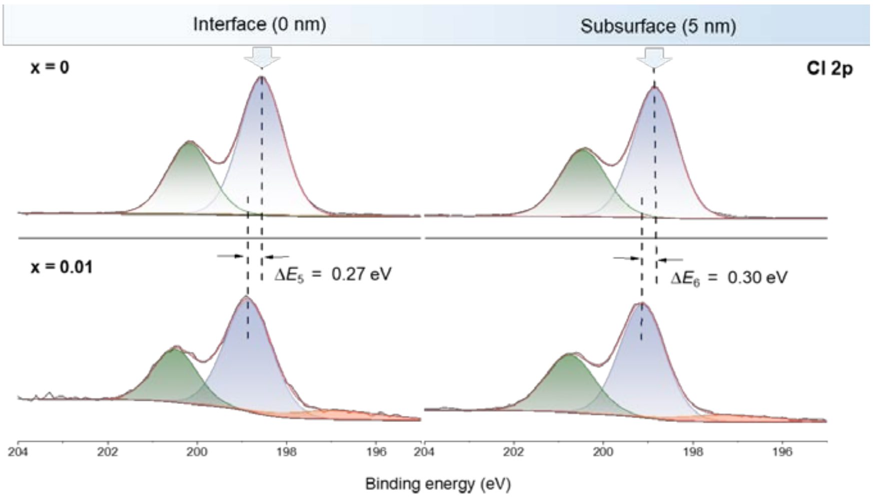

Figure S37 Cl 2p XPS spectra of $Li_{5.5-x}P_{1-x}W_xS_{4.5-3x}O_{3x}Cl_{1.5}$ SSE-based composite cathodes on surface and subsurface.

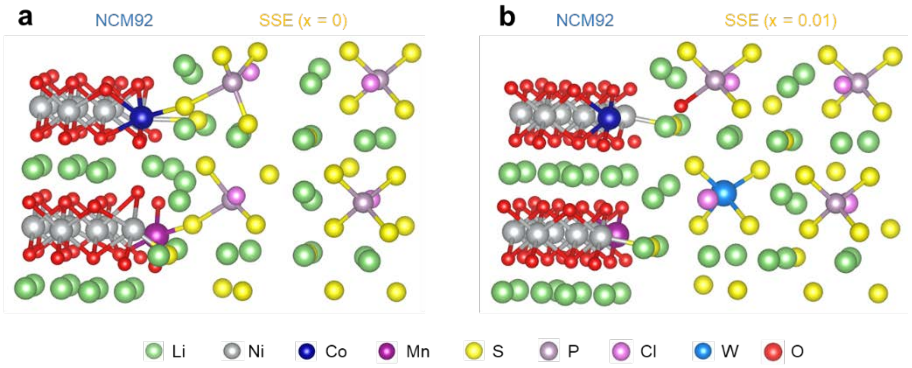

Figure S38 Crystalline structures of cathodic interfaces with $Li_{5.5-x}P_{1-x}W_xS_{4.5-3x}O_{3x}Cl_{1.5}$ SSEs. (a) $x = 0$, (b) $x = 0.01$.

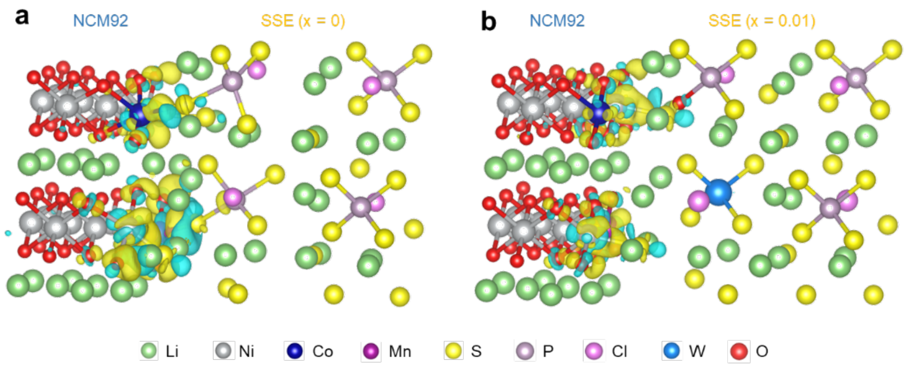

Figure S39 Differential charge densities of cathodic interfaces with $Li_{5.5\text{-}x}P_{1\text{-}x}W_xS_{4.5\text{-}3x}O_{3x}Cl_{1.5}$ SSEs. (a) $x = 0$, (b) $x = 0.01$.

Supplementary Tables

Table S1. Structural parameters for $Li_{5.49}P_{0.99}W_{0.01}S_{4.47}O_{0.03}Cl_{1.5}$ SSE from Rietveld refinement analysis of XRD data collected at T = 298 K. [a]

**Atom**	**Wyckoff site**	**x**	**y**	**z**	**Occ.**	U_{iso}
Li1	48h	0.898	0.388554	0.602	0.375	0.01
P1	4b	0.5	0.5	0.5	0.9715	0.0234
W1	4b	0.5	0.5	0.5	0.0885	0.0234
S1	4a	0	0	0	0.21	0.0592
Cl1	4a	0	0	0	0.253	0.0592
Br1	4a	0	0	0	0.553	0.0592
S2	4b	0.25	0.25	0.25	0.289	0.025
Cl2	4b	0.25	0.25	0.25	0.555	0.025
Br2	4b	0.25	0.25	0.25	0.147	0.025
S3	16e	0.62315	0.62315	0.62315	0.925	0.0039
O1	16e	0.62315	0.62315	0.62315	0.075	0.0039

[a] *F-43m* space group, a = 9.87654, V = 963.417Å^3, R_{wp} = 10.9%, GOF = 1.48.

Table S2. Structural parameters for $Li_{5.5}S_{4.5}Cl_{1.5}$ SSE from Rietveld refinement analysis of XRD data collected at T = 298 K. [a]

**Atom**	**Wyckoff site**	**x**	**y**	**z**	**Occ.**	U_{iso}
Li1	48h	0.817	0.476	0.683	0.53	0.12
P1	4b	0.5	0.5	0.5	0.9715	0.025
S1	4a	0	0	0	0.21	0.00649
Cl1	4a	0	0	0	0.253	0.01
S2	4b	0.25	0.25	0.25	0.289	0.025
Cl2	4b	0.25	0.25	0.25	0.555	0.01
S3	16e	0.6217	0.6217	0.6217	0.925	0.02171

[a] *F-43m* space group, a = 9.78943, V = 938.218$Å^3$, R_{wp} = 9.99%, GOF = 3.89.

Table S3. σ_i and E_a of prepared SSEs at 25 °C.

$Li_{5.5-x}P_{1-x}W_xS_{4.5-3x}O_{3x}Cl_{1.5}$	Thickness (cm)	Total resistance (Ω)	σ_i (mS cm^{-1})	E_a (eV)
0	0.097	14.0	8.8	0.234
0.005	0.095	13.6	8.9	0.208
0.01	0.096	9.0	13.5	0.191
0.015	0.094	15.8	7.6	0.244

Table S4. σ_i, E_a and σ_e of previously reported O-containing argyrodite electrolytes at 25 °C.

O-containing argyrodite SSEs	σ_i of SSE matrix (mS cm^{-1})	σ_i of substituted SSEs (mS cm^{-1})	E_a (eV)	σ_e (S cm^{-1})	Ref.
$Li_{6.1}P_{0.95}Y_{0.05}S_{4.925}O_{0.075}Cl$	2.75	**3.53**	0.34	1.55×10^{-7}	[3]
$Li_{6.16}P_{0.92}In_{0.08}S_{4.88}O_{0.12}Cl$	1.768	**2.67**	0.278	1.876×10^{-9}	[4]
$Li_{6}P_{0.95}Sb_{0.05}S_{4.875}O_{0.125}Cl$	--	**5.3**	0.25	2.06×10^{-9}	[5]
$Li_{6.05}P_{0.95}Mo_{0.05}S_{4.9}O_{0.1}Cl$	5.2	**4.16**	0.299	2.04×10^{-8}	[6]
$Li_{6.04}P_{0.98}Bi_{0.02}S_{4.97}O_{0.03}Cl$	2.4	**3.4**	0.261	5.3×10^{-9}	[7]
$Li_{5.5}P_{0.9}Sn_{0.1}S_{4.2}O_{0.2}Cl_{1.6}$	8.2	**8.7**	0.18	--	[8]
$Li_{5.7}P_{0.9}Nb_{0.1}S_{4.65}O_{0.05}Cl_{1.3}$	4.52	**6.95**	0.34	--	[9]
$Li_{5.5}P_{0.95}Nb_{0.05}S_{4.375}O_{0.125}Cl_{1.5}$	7	**4.5**	0.26	--	[10]
$Li_{6.05}P_{0.95}Zr_{0.05}S_{4.9}O_{0.1}Cl$	3.62	**3.97**	0.273	6.11×10^{-10}	[11]
$Li_{5.5}P_{0.96}Sb_{0.04}S_{4.40}O_{0.10}Cl_{1.5}$	9.0	**7.20**	--	--	[12]
$Li_{5.95}Zn_{0.025}PS_{4.975}O_{0.025}Cl$	4.55	**4.08**	0.299	--	[13]
$Li_{5.51}P_{0.99}Mo_{0.01}S_{4.48}O_{0.02}Cl_{1.5}$	9.1	**12.0**	0.28	1.27×10^{-8}	[14]
**$Li_{5.49}P_{0.99}W_{0.01}S_{4.47}O_{0.03}Cl_{1.5}$**	**8.8**	**13.5**	**0.19**	**4.58×10^{-9}**	**This work**

Table S5. Measured σ_i (mS cm^{-1}) of $Li_{5.5-x}P_{1-x}W_xS_{4.5-3x}O_{3x}Cl_{1.5}$ SSEs in the temperature range of 25 ℃ to 70 ℃.

$Li_{5.5-x}P_{1-x}W_xS_{4.5-3x}O_{3x}Cl_{1.5}$	25 ℃	40 ℃	50 ℃	60 ℃	70 ℃
0	8.8	13.4	17.1	22.0	27.7
0.005	8.9	13.0	16.1	20.0	24.3
0.010	13.5	18.8	22.9	28.0	33.3
0.015	7.6	11.7	15.5	19.8	25.1

Table S6. Measured σ_e of $Li_{5.5-x}P_{1-x}W_xS_{4.5-3x}O_{3x}Cl_{1.5}$ SSEs from current response after applying a DC voltage at 25 °C.

$Li_{5.5-x}P_{1-x}W_xS_{4.5-3x}O_{3x}Cl_{1.5}$	σ_e (S cm^{-1})
0	6.87×10^{-8}
0.005	5.73×10^{-9}
0.01	4.58×10^{-9}
0.015	1.63×10^{-8}

Table S7. Summary of the electrochemical performance of Li–In|NCM full cells with Cl-rich argyrodite SSEs.

SSE	Cathode	Loading of cathodes (mg cm^{-2})	Rate (C)	Voltage range (V vs. Li/Li^{+})	Capacity (mAh g^{-1})	Stability Cycle/capacity retention	Ref.
$Li_{5.5}PS_{4.425}O_{0.075}Cl_{1.5}$	NCM811	2.55	0.2	3-4.3	152.4	100/95.1%	[15]
$Li_{5.5}P_{0.9}Sn_{0.1}S_{4.2}O_{0.2}Cl_{1.6}$	NCM811	5	0.5	2.2-3.7	119.1	100/74.7%	[8]
$Li_{5.65}Si_{0.05}Ge_{0.05}Sn_{0.05}P_{0.85}S_{4.5}Cl_{0.5}Br$	NCM712@Nb	4.46	3	3-4.3	134.2	900/79.2%	[16]
$Li_{5.5}PS_{4.5}Cl_{1.5}$	s-NCM90@Li_3BO_3	8.9	2	2.4-3.7	108	700/99.5%	[17]
$Li_{5.45}Ag_{0.05}PS_{4.5}Cl_{1.5}$	NCM622	3.82	1	2.4-3.7	154.9	100/94.5%	[18]
$Li_{5.5}P_{0.96}Sb_{0.04}S_{4.40}O_{0.10}Cl_{1.5}$	NCM622	4.46	0.1	2.4-3.7	162.4	80/85.6%	[12]
$Li_{5.7}PS_{4.7}Cl_{1.3}$	$LiNbO_3$@ $LiNi_{0.8}Mn_{0.1}Co_{0.1}O_2$	8.92	2	1.88-3.78	83.4	100/59.23%	[19]
$Li_{5.7}PS_{4.7}Cl_{1.3}$	LNO@NCM622	4	0.5	2-3.7	148	1000/94%	[20]
$Li_{5.5}PS_{4.5}Cl_{1.5}$	NCM811	6.24	0.2	2.5-4.25	151.4	100/80.4%	[21]
$Li_{5.5}PS_{4.5}Cl_{1.5}$	NCM85	15.28	0.5	2.6-4.3	170	50/85%	[22]
**$Li_{5.49}P_{0.99}W_{0.01}S_{4.47}O_{0.03}Cl_{1.5}$**	**NCM92**	**8.92**	**1**	**2.5-3.7**	**172**	**1000/92%**	**This work**

Table S8. Summary of the limited areal capacity of full cells with argyrodite SSEs and Li–In anode.

**SSE**	**Cathode**	**Loading of cathodes (mg cm^{-2})**	**Rate**	**Areal Capacity (mAh cm^{-2})**	**Ref.**
Li_6PS_5Cl	$Li[Ni_{0.9}Co_{0.05}Mn_{0.05}]O_2$	23.04	0.1 C	4.25	[23]
$Li_{5.5}(P_{0.9}Sn_{0.1})(S_{4.2}O_{0.2})Cl_{1.6}$	$LiNi_{0.8}Co_{0.1}Mn_{0.1}O_2$	5	0.1 C	0.6	[8]
$Li_6PS_5Cl_{0.5}Br_{0.5}$	$Li_6PS_5Cl_{0.5}Br_{0.5}$ -MWCNTs composite	24.8	0.01C	12.56	[24]
Li_6PS_5Cl@P(VDF-TrFE)	$LiNi_{0.8}Co_{0.1}Mn_{0.1}O_2$ @LNO	24.78	0.161C	3.05	[25]
Li_6PS_5Cl	$LiNi_{0.8}Co_{0.1}Mn_{0.1}O_2$	35.7	2 C	6.43	[26]
$Li_{6.8}Si_{0.8}As_{0.2}S_5I$	TiS_2	44.56	0.035 C	9.26	[27]
$Li_{6.8}Si_{0.8}As_{0.2}S_5I$	FeS_2	13.37	0.05C	9.05	[28]
Li_6PS_5Cl with EMG	$LiNi_{0.9}Co_{0.05}Mn_{0.05}O_2$	24	0.05C	5.5	[29]
**$Li_{5.49}P_{0.99}W_{0.01}S_{4.47}O_{0.03}Cl_{1.5}$**	**NCM92**	**101.91**	**0.1C**	**16.1**	**This work**

Table S9. The fitted resistances of full cells with different SSEs after various cycles.

Cycle number	$Li_{5.5}PS_{4.5}Cl_{1.5}$		$Li_{5.49}P_{0.99}W_{0.01}S_{4.47}O_{0.03}Cl_{1.5}$	
	R_{bulk} (Ω)	$R_{interface}$ (Ω)	R_{bulk} (Ω)	$R_{interface}$ (Ω)
0 cycle	20.00	27.73	17.8	23.40
300 cycles	11.25	544.40	21.01	20.37
500 cycles	11.77	494.10	20.98	21.38
1000 cycles	12.80	552.6	12.27	38.89

Table S10. Fitted resistances of full cells with two SSEs under various voltages during charging.

$Li_{5.5-x}P_{1-x}W_xS_{4.5-3x}O_{3x}Cl_{1.5}$	Voltage (V)	R_{bulk}	R_{a-i}	R_{c-i}
x = 0.01	2.5	22.87	10.48	64.54
	2.8	21.97	10.90	61.28
	3.1	21.96	13.54	57.18
	3.4	22.59	17.76	59.10
	3.7	20.72	20.42	48.39
x = 0	2.5	23.22	28.07	95.46
	2.8	22.91	29.71	85.93
	3.1	23.09	31.91	82.13
	3.4	23.06	34.38	84.70
	3.7	23.51	65.38	103.85

Table S11. The calculated energy density of the pouch cell with Li/μ-Si anode, NCM92 CAM, and $Li_{5.49}P_{0.99}W_{0.01}S_{4.47}O_{0.03}Cl_{1.5}$ SSE.

Assembly	Items	Values
Cathode	Specific capacity	191 mAh g^{-1}
	CAM loading	29 mg cm^{-2}
	CAM content	70 wt%
	Area	100 cm^2
SSE	Thickness	65 μm
	Weight	0.78 g
Anode	Weight	0.42 g
Pouch cell	Voltage	3.5 V
	Capacity	554 mAh
	Weight	5.34 g
	Gravimetric energy density	**363 Wh kg^{-1}**

Note: 1. The specific capacity is based on 0.2C;

2. The energy density is calculated only based on total weight of cathode, electrolyte and anode.

References

1. Jung WD, Kim J-S, Choi S *et al.* Superionic halogen-rich Li-argyrodites using in situ nanocrystal nucleation and rapid crystal growth. *Nano Lett* 2020; **20**: 2303-2309.

2. Adeli P, Bazak JD, Park KH *et al.* Boosting solid-state diffusivity and conductivity in lithium superionic argyrodites by halide substitution. *Angew Chem Int Ed* 2019; **58**: 8681-8686.

3. Wang D, Liu C, Wang R *et al.* Electronic localization enables long-cycling sulfides-based all-solid-state lithium batteries. *Angew Chem Int Ed* 2025; **64**: e202501411.

4. Wang C, Hao J, Wu J *et al.* Enhanced air stability and Li metal compatibility of Li-argyrodite electrolytes triggered by In_2O_3 co-doping for all-solid-state Li metal batteries. *Adv Funct Mater* 2024; **34**: 2313308.

5. Liu C, Zhang T, Wang R *et al.* Regulating p-band center of sulfur in Li-argyrodite to stabilize dual solid–solid interface for robust all-solid-state lithium–sulfur battery. *Adv Funct Mater* 2025; **35**: 2412144.

6. Subramanian Y, Rajagopal R, Kang S *et al.* Enhancement of lithium argyrodite interface stability through MoO_2 substitution and its application in lithium solid state batteries. *J Alloys Compd* 2022; **925**: 166596.

7. Liu H, Zhu Q, Wang C *et al.* High air stability and excellent Li metal compatibility of argyrodite-based electrolyte enabling superior all-solid-state Li metal batteries. *Adv Funct Mater* 2022; **32**: 2203858.

8. Li G, Wu S, Zheng H *et al.* Sn-O dual-substituted chlorine-rich argyrodite electrolyte with enhanced moisture and electrochemical stability. *Adv Funct Mater* 2023; **33**: 2211805.

9. Li S, Lin Q, Yan H *et al.* Enhanced air stability and interface compatibility in Nb-O-doped Cl-rich Li-argyrodites for all-solid-state Li metal batteries. *Electrochim Acta* 2025; **509**:145341.

10. Khan AJ, Ding H, Fu C *et al.* Argyrodite $Li_{5.5}(P_{1-x}Nb_x)(S_{4.5-2.5x}O_{2.5x})Cl_{1.5}$ solid electrolytes with enhanced electrochemical stability for all-solid-state batteries. *J Alloys Compd* 2025; **1041**: 183892.

11. Wang D, Shi H, Cui W *et al.* Li-argyrodite solid-state electrolytes with lithium compatibility

and air stability for all-solid-state batteries. *J Mater Chem A* 2024; **12**: 10863-10874.

12. Wei C, Yu C, Wang R *et al.* Sb and O dual doping of chlorine-rich lithium argyrodite to improve air stability and lithium compatibility for all-solid-state batteries. *J Power Sources* 2023; **559**: 232659.

13. Jang G-J, Rajagopal R, Kang S *et al.* Preparation of argyrodite $Li_{6-2x}Zn_xPS_{5-x}O_xCl$ with improved electrochemical performance and air stability for all-solid-state batteries. *J Alloys Compd* 2023; **957**: 170273.

14. Wu Y, Zhang R, Huang Q *et al.* A highly conductive and antioxidative MoO_2-doped Li argyrodite electrolyte for all-solid-state Li batteries. *Chem Commun* 2024; **60**: 13578-13581.

15. Peng L, Chen S, Yu C *et al.* Enhancing moisture and electrochemical stability of the Li(5.5)PS(4.5)Cl(1.5) electrolyte by oxygen doping. *ACS Appl Mater Interfaces* 2022; **14**: 4179–4185.

16. Li W, Chen Z, Chen Y *et al.* High-entropy argyrodite-type sulfide electrolyte with high conductivity and electro-chemo-mechanical stability for fast-charging all-solid-state batteries. *Adv Funct Mater* 2024; **34**: 2312832.

17. Li S, Lin J, Schaller M *et al.* High-entropy lithium argyrodite solid electrolytes enabling stable all-solid-state batteries. *Angew Chem Int Ed* 2023; 62: e202314155.

18. Wu Z, Yu C, Wei C *et al.* Ag-modification argyrodite electrolytes enable high-performance for all-solid-state lithium metal batteries. *Chem Eng J* 2023; **466**: 143304.

19. Yu C, Li Y, Willans M *et al.* Superionic conductivity in lithium argyrodite solid-state electrolyte by controlled Cl-doping. *Nano Energy* 2020; **69**: 104396.

20. Zeng D, Yao J, Zhang L *et al.* Promoting favorable interfacial properties in lithium-based batteries using chlorine-rich sulfide inorganic solid-state electrolytes. *Nat Commun* 2022; **13**(1): 1909.

21. Liu Y, Su H, Zhong Y *et al.* Revealing the impact of Cl substitution on the crystallization behavior and interfacial stability of superionic lithium argyrodites. *Adv Funct Mater* 2022; **32**: 2207978.

22. Zuo T-T, Walther F, Teo JH *et al.* Impact of the chlorination of lithium argyrodites on the electrolyte/cathode interface in solid-state batteries. *Angew Chem Int Ed* 2023; **62**: e202213228.

23. Kim U-H, Yu T-Y, Lee JW *et al.* Microstructure- and interface-modified Ni-rich cathode for

high-energy-density all-solid-state lithium batteries. *ACS Energy Lett* 2023; **8**: 809-817.

24. Wang S, Tang M, Zhang Q *et al.* Lithium argyrodite as solid electrolyte and cathode precursor for solid-state batteries with long cycle life. *Adv Energy Mater* 2021; **11**: 2101370.

25. Liu S, Zhou L, Han J *et al.* Super long-cycling all-solid-state battery with thin Li_6PS_5Cl-based electrolyte. *Adv Energy Mater* 2022; **12**: 2200660.

26. Liang Z, Xiao Y, Wang K *et al.* Enabling stable and high areal capacity solid state battery with Ni-rich cathode via failure mechanism study. *Energy Storage Mater* 2023; **63**: 102987.

27. Lu P, Xia Y, Sun G *et al.* Realizing long-cycling all-solid-state Li-In||TiS_2 batteries using $Li_{6+x}M_xAs_{1-x}S_5I$ (M=Si, Sn) sulfide solid electrolytes. *Nat Commun* 2023; **14**: 4077.

28. Lu P, Xia Y, Huang Y *et al.* Wide-temperature, long-cycling, and high-loading pyrite all-solid-state batteries enabled by argyrodite thioarsenate superionic conductor. *Adv Funct Mater* 2023; **33**: 2211211.

29. Hong S-B, Jang Y-R, Jung Y-C *et al.* Sulfide-based flexible solid electrolyte enhancing cycling performance of all-solid-state lithium batteries. *ACS Appl Energy Mater* 2024; **7**: 5193-5201.
